# Laminin regulates PDGFRβ^+^ cell stemness and muscle development

**DOI:** 10.1038/ncomms11415

**Published:** 2016-05-03

**Authors:** Yao Yao, Erin H. Norris, Christopher E. Mason, Sidney Strickland

**Affiliations:** 1 Laboratory of Neurobiology and Genetics, The Rockefeller University, 1230 York Avenue, New York, New York 10065, USA; 2 College of Pharmacy, University of Minnesota, 1110 Kirby Drive, Duluth, Minnesota 55812, USA; 3 Department of Physiology and Biophysics, Weill Cornell Medical College, New York, New York 10065, USA; 4 The HRH Prince Alwaleed Bin Talal Bin Abdulaziz Alsaud Institute for Computational Biomedicine, New York, New York 10065, USA; 5 Tri-Institutional Training Program in Computational Biology and Medicine, New York, New York 10065, USA; 6 The Feil Family Brain and Mind Research Institute, New York, New York 10065, USA

## Abstract

Muscle-resident PDGFRβ^+^ cells, which include pericytes and PW1^+^ interstitial cells (PICs), play a dual role in muscular dystrophy. They can either undergo myogenesis to promote muscle regeneration or differentiate into adipocytes and other cells to compromise regeneration. How the differentiation and fate determination of PDGFRβ^+^ cells are regulated, however, remains unclear. Here, by utilizing a conditional knockout mouse line, we report that PDGFRβ^+^ cell-derived laminin inhibits their proliferation and adipogenesis, but is indispensable for their myogenesis. In addition, we show that laminin alone is able to partially reverse the muscle dystrophic phenotype in these mice at the molecular, structural and functional levels. Further RNAseq analysis reveals that laminin regulates PDGFRβ^+^ cell differentiation/fate determination via gpihbp1. These data support a critical role of laminin in the regulation of PDGFRβ^+^ cell stemness, identify an innovative target for future drug development and may provide an effective treatment for muscular dystrophy.

## Abstract (web summary)

Muscle PDGFRβ^+^ cells are interstitial stem/progenitor cells with myogenic potential. Here, Yao *et al*. show that PDGFRβ^+^ cell-derived laminin actively regulates their proliferation, differentiation and fate determination.

## Graphical abstract

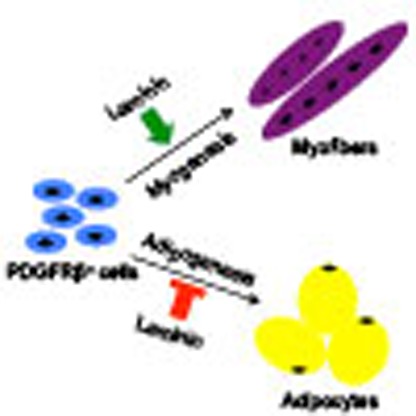

Muscular dystrophy (MD) is a genetic disorder characterized by progressive degeneration and weakness of muscles. Congenital muscular dystrophy (CMD), a severe type of MD, usually has its onset at or near birth and affects almost all the voluntary muscles in the body^1^. Although physical therapy and other medical management have been found beneficial, there are no effective treatments for this devastating disorder. Stem cells with myogenic activity have been suggested as a promising therapy for MD. Satellite cells, postnatal myogenic precursor cells, demonstrate great potential by actively repairing muscle damage and promoting regeneration after injury^2^,^3^,^4^,^5^,^6^,^7^. Their clinical application, however, is hampered by their limited migration ability^8^, low survival rate after injection^9^,^10^ and reduced differentiation potency after expansion^11^.

In addition to satellite cells, muscle-resident PDGFRβ^+^ cells also have myogenic activity. Using lineage-tracing technique, we found that PDGFRβ^+^ cells include two populations: pericytes and PICs. There is evidence showing that pericytes, multipotent perivascular cells^12^, can differentiate into myogenic cells and repair damage after muscle injury^12^,^13^,^14^,^15^,^16^. Pericytes, on the other hand, can also differentiate into adipocytes, which contribute to muscle degeneration. It has been demonstrated that PICs are myogenic *in vitro* and contribute to skeletal muscle regeneration efficiently *in vivo*^17^,^18^,^19^,^20^. These data suggest that PDGFRβ^+^ cells may be used to treat MD. How PDGFRβ^+^ cell stemness (self-renewal, differentiation and fate determination) is regulated remains unknown. Under physiological conditions, these cells are embedded in the basement membrane^21^, which usually gets degraded upon injury. This direct contact suggests that the basement membrane may regulate the stemness of PDGFRβ^+^ cells.

Laminin, a trimeric protein containing α-, β- and γ-subunits, is a major component of the basement membrane. Loss of laminin α2 (*Lama2*) subunit leads to progressive MD and has been used as a mouse model of CMD^22^,^23^,^24^,^25^. Recent research demonstrates that laminin-α1β1γ1 (laminin-111) is able to reduce muscle pathology, enhance viability and improve muscle repair in a *Lama2*-deficient CMD model^26^,^27^. In addition, laminin-111 also significantly improves muscle pathology and increases muscle strength/resistance in mdx mice^28^,^29^, a Duchenne muscular dystrophy model. These data strongly suggest a therapeutic function of laminin in MD. The mechanism underlying laminin's beneficial role in CMD, however, remains unclear. Previous studies in our lab showed that (i) astrocyte-derived laminin maintains the blood–brain barrier integrity by preventing brain pericytes differentiating from the resting stage to the contractile stage^30^ and (ii) aortic stem/progenitor cell-derived laminin modulates their proliferation and differentiation, contributing to blood pressure regulation^31^. Given that PDGFRβ^+^ cells also produce laminin that covers themselves in skeletal muscles, we hypothesized that PDGFRβ^+^ cell-derived laminin regulates their stemness in skeletal muscles.

By using conditional knockout mice with laminin deficiency in PDGFRβ^+^ cells and RNAseq analysis, we show that (i) PDGFRβ^+^ cell-derived laminin negatively regulates their proliferation and adipogenesis, but promotes their myogenesis, (ii) gpihbp1 is an important mediator of these effects and (iii) laminin-111 is able to partially rescue the muscle phenotype in these mice at molecular, structural and functional levels.

## Results

### Loss of PDGFRβ^+^ cell-derived laminin leads to MD

**Figure 1:**
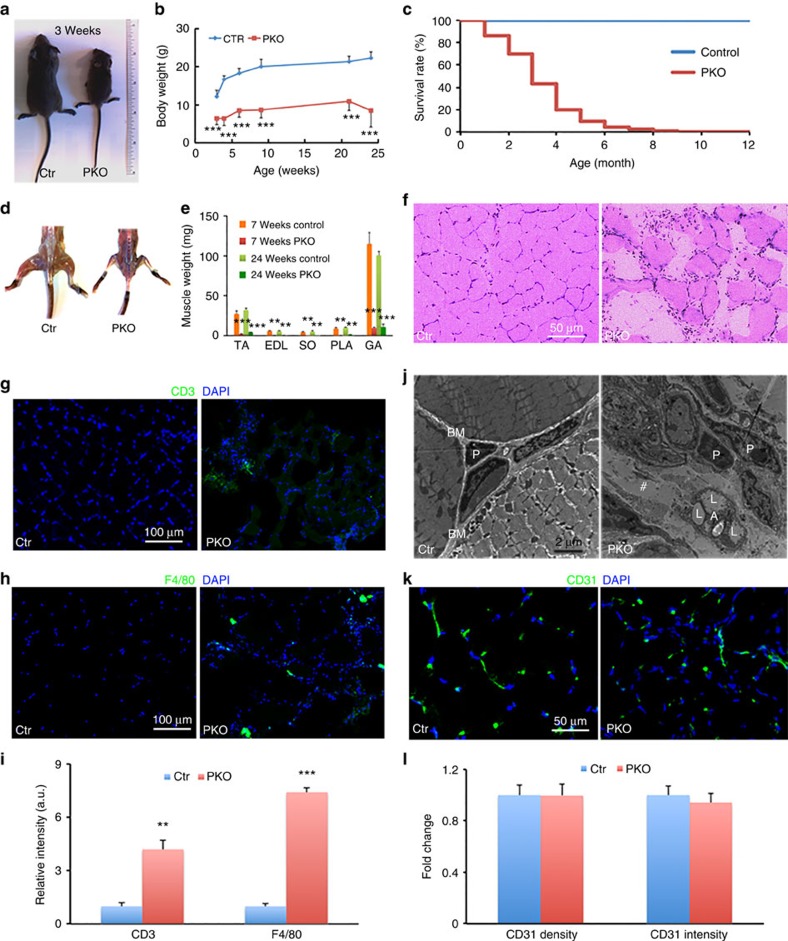
The PKO phenotype. (**a**) Size comparison of 3-week-old control and PKO mice. (**b**) Body weight quantification over time; *n*=10 for 3–6 weeks, 6 for 9 weeks and 4 for 21–24 weeks. (**c**) Survival rate of control and PKO mice over time. (**d**) Hindlimb muscle comparison between control and PKO mice. (**e**) Quantification of individual hindlimb muscle weight at 7 and 24 weeks; *n*=5. (**f**) H&E staining of hindlimb muscles. (**g**,**h**) Immunohistochemical analyses of CD3 (**g**, green) and F4/80 (**h**, green) infiltration in control and PKO muscles. (**i**) Quantification of CD3 and F4/80 expression in control and PKO muscles; *n*=3. (**j**) Representative ultrastructural images of hindlimb muscles. A, adipocytes; BM, basement membrane; L, lipid droplets; P, pericytes; #, gap. (**k**) Immunohistochemical analyses of CD31 (green) expression in control and PKO muscles. (**l**) Quantification of CD31 expression; *n*=3. Scale bars, 50 μm in **f** and **k**; 100 μm in **g** and **h**; and 2 μm in **j**. **P<0.01; ***P<0.001 (Student's *t*-test). The results are shown as mean±s.d.

We generated homozygous Laminin γ1 (*Lamc1*) floxed (F/F) mice by targeting the exon 2 of *Lamc1* gene as described previously^32^. The F/F mice were then crossed with *Pdgfrβ-Cre* transgenic mice to generate F/F:*Pdgfrβ-Cre*^*+*^ (termed PKO) mice. The PKO mice were born at the Mendelian ratio and were indistinguishable from their heterozygous and wild-type littermates at early postnatal stage. Starting from approximately postnatal day (P)10, the PKO mice became significantly smaller than their littermate controls (Fig. 1a,b). The PKO mice usually died within 4 months as demonstrated by their survival rate (Fig. 1c). In addition, the PKO mice developed a severe skeletal muscle deficit (Fig. 1d), similar to that in *Lama2*-deficient mice, a widely used genetic model of CMD. All hindlimb muscles, including tibialis anterior, extensor digitorum longus, soleus, plantaris and gastrocnemius, were significantly smaller in the PKO mice 7 and 24 weeks after birth (Fig. 1e). Haematoxylin and eosin (H&E) staining revealed well-developed myofibres in 2-month-old control muscles (Fig. 1f). The myofibres in age-matched PKO muscles, however, varied in size and contained centrally nucleated nuclei (Fig. 1f), suggesting active muscle degeneration/regeneration. Like the *Lama2*-deficient mice^33^,^34^,^35^,^36^,^37^, the PKO mice also showed signs of muscle necrosis and fibrosis in pathological analyses. In addition, a large number of nuclei were observed in the interstitium of the PKO muscles (Fig. 1f). Further immunohistochemical analyses revealed significantly increased infiltration of T cells (Fig. 1g,i) and macrophages (Fig. 1h,i) in the PKO muscles, compared with the controls. Inflammatory cell infiltration, a hallmark of CMD^38^, is also observed in the *Lama2*-deficient mice^33^,^35^,^36^. Consistent with these changes, electron microscopy study revealed well-developed myofibres wrapped by a clearly defined basement membrane in control mice (Fig. 1j). In the PKO muscles, however, well-developed myofibres and a clearly defined basement membrane were missing and were instead replaced by gaps (#, Fig. 1j) containing adipocytes with lipid droplets. In addition, we also performed CD31 staining and found no differences in CD31 density or intensity between the control and PKO mice (Fig. 1k,l), suggesting that the observed phenotype is less likely due to vascular defects. Interestingly, the muscle pathology (Supplementary Fig. 1a and b) and inflammatory cell infiltration (Supplementary Fig. 1c) were absent in the PKO mice at P6. A time-course study showed that the PKO phenotype started at approximately P10. Previous study demonstrated that in the *Lama2*-deficient mice, muscle pathology appeared at P9 and became apparent at P14 (ref. 33). Collectively, our data demonstrate that the gross phenotype, muscle pathology and timing of disease onset/development are comparable between the PKO mice and *Lama2*-deficient mice, suggesting that the PKO mice may be used to study CMD.

**Figure 2:**
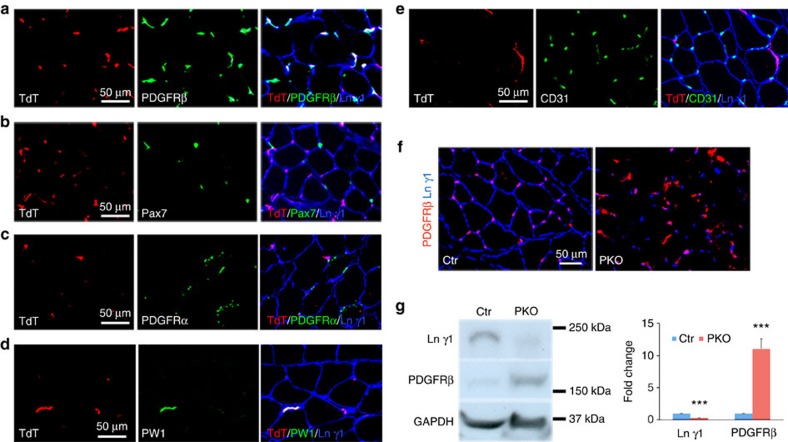
Specificity of *Pdgfrβ*-driven *Cre*. (**a**–**e**) TdT (red) expression co-localized with PDGFRβ (**a**, green), PW1 (**d**, green), but not Pax7 (**b**, green), PDGFRα (**c**, green) or CD31 (**e**, green) in Ai14:*Pdgfrβ-Cre*^+^ reporter mice. TdT, tdTomato. (**f**) Laminin γ1 (blue) and PDGFRβ (red) expression in tibialis anterior muscles. (**g**) Western blot analysis of laminin γ1 and PDGFRβ expression in skeletal muscles. GAPDH was used as a loading control; *n*=4. Scale bars, 50 μm. ***P<0.001 (Student's *t*-test). The results are shown as mean±s.d.

To investigate the specificity of *Pdgfrβ*-driven *Cre*, the *Pdgfrβ-Cre* mice were crossed with the Ai14 reporter line, which contains a floxed STOP sequence before tdTomato (TdT). In the resulting Ai14:*Pdgfrβ-Cre*^+^ mice, TdT expression co-localized with pericyte marker PDGFRβ (Fig. 2a). Satellite cell maker Pax7 (Fig. 2b), fibro/adipogenic progenitor (FAP) marker PDGFRα (Fig. 2c) and endothelial marker CD31 (Fig. 2e) rarely co-localized with TdT, suggesting that *Lamc1* is not targeted in these cells. Interestingly, PW1, a marker for PICs, co-localized with TdT (Fig. 2d), suggesting that *Lamc1* in PICs is also targeted. In addition, we also examined the expression pattern of these markers with TdT in F/F:Ai14:*Pdgfrβ-Cre*^+^ mice and similar results were found (Supplementary Fig. 2a-e). In these mice, hematopoietic (CD45^+^) cells infiltrated into the muscles and they were not labelled by TdT (Supplementary Fig. 2f), suggesting that the floxed allele is not targeted in these CD45^+^ cells. Consistent with these data, we found dramatically reduced laminin γ1 expression in PDGFRβ^+^ cells in the PKO muscles (Fig. 2f). The western blots showed similar results (Fig. 2g).

**Figure 3:**
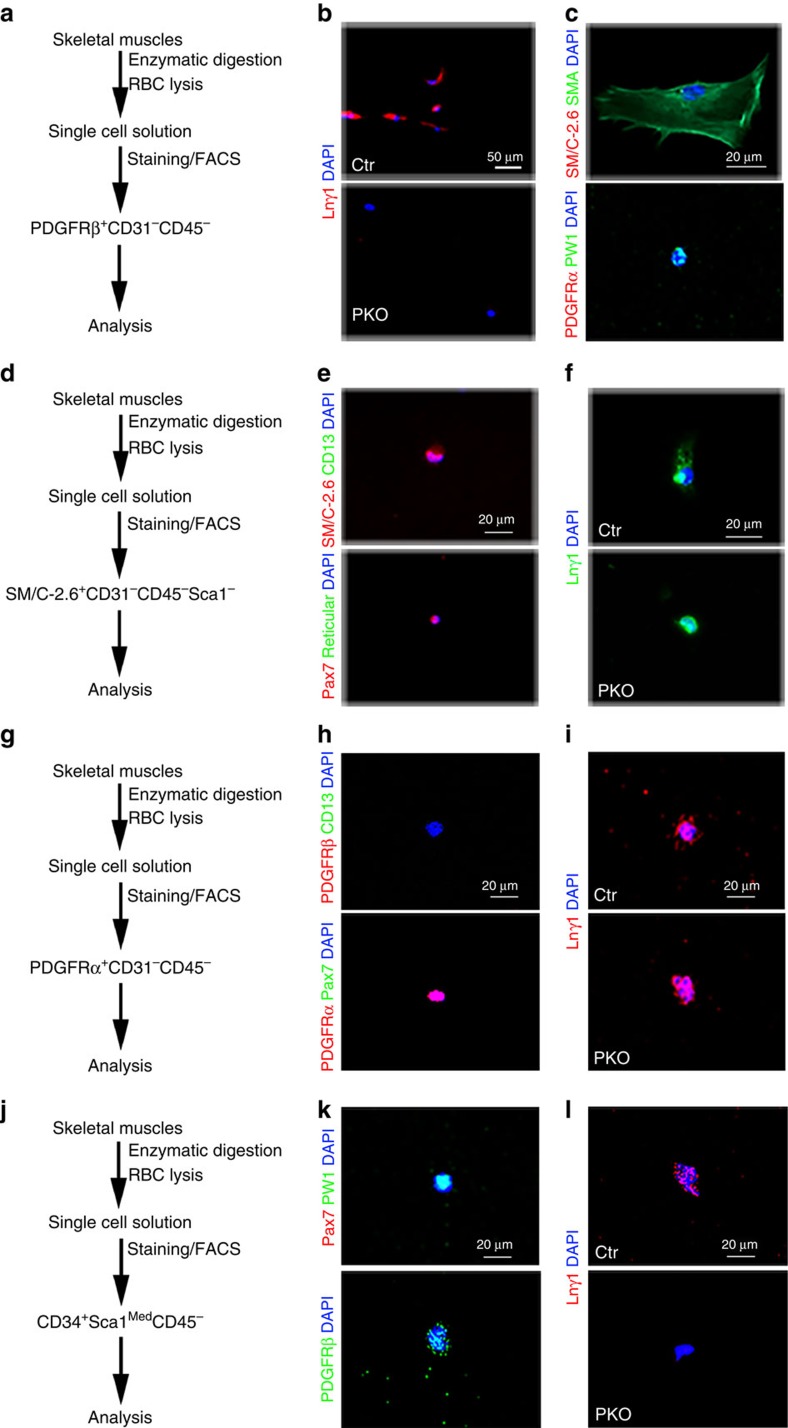
Laminin expression in sorted primary cells. (**a**) Schematic illustration of PDGFRβ^+^ cell-isolating procedure. (**b**) Laminin γ1 (red) expression in PDGFRβ^+^ cells isolated from control and PKO mice. (**c**) SM/C-2.6 (red)/SMA (green) and PDGFRα (red)/PW1 (green) expression in wild-type PDGFRβ^+^ cells. (**d**) Schematic illustration of satellite cell-isolating procedure. (**e**) SM/C-2.6 (red)/CD13 (green) and Pax7 (red)/Reticular (green) expression in wild-type satellite cells. (**f**) Laminin γ1 (green) expression in satellite cells isolated from control and PKO mice. (**g**) Schematic illustration of FAP-isolating procedure. (**h**) PDGFRβ (red)/CD13 (green) and PDGFRα (red)/Pax7 (green) expression in wild-type FAPs. (**i**) Laminin γ1 (red) expression in FAPs isolated from control and PKO mice. (**j**) Schematic illustration of PIC-isolating procedure. (**k**) Pax7 (red)/PW1 (green) and PDGFRβ (green) expression in wild-type PICs. (**l**) Laminin γ1 (red) expression in PICs isolated from control and PKO mice. Scale bars, 50 μm in **b** and 20 μm in **c**,**e**,**f**,**h**,**i**,**k** and **l**.

Furthermore, we isolated PDGFRβ^+^ cells from skeletal muscles by FACS^12^,^13^,^16^ (Fig. 3a) and examined laminin γ1 expression on these cells. Fluorescence-minus-one control for PDGFRβ-PE was used to set its gating (Supplementary Fig. 3a). Laminin γ1 expression was observed in sorted control but not the PKO cells (Fig. 3b), indicating that laminin expression is indeed diminished in PDGFRβ^+^ cells in the PKO mice. To determine the cell types in PDGFRβ^+^ population, we checked the expression of various markers on these cells. Sorted PDGFRβ^+^ cells expressed either pericyte markers, including NG2, CD13, SMA and alkaline phosphatase (ALP) or PIC marker PW1 (Fig. 3c and Supplementary Fig. 4a and b). These PDGFRβ^+^ cells, however, were negative for satellite cell marker SM/C-2.6 and FAP marker PDGFRα (Fig. 3c), suggesting that sorted PDGFRβ^+^ cells contain pericytes and PICs, but not satellite cells or FAPs. In addition, we also isolated satellite cells^39^,^40^, FAPs^41^,^42^,^43^ and PICs^17^ from the control and PKO muscles by FACS (Fig. 3d,g,j). Representative plots and gating boundaries were shown in Supplementary Fig. 3b–d. Sorted satellite cells expressed SM/C-2.6 and Pax7, but were negative for PDGFRβ, CD13, PDGFRα and fibroblast marker Reticular (Fig. 3e and Supplementary Fig. 4c and d). Comparable levels of laminin γ1 were observed in satellite cells from the control and PKO mice (Fig. 3f), suggesting that laminin expression is not affected in satellite cells in the PKO mice. Sorted FAPs expressed PDGFRα, but were negative for PDGFRβ, CD13 and Pax7 (Fig. 3h). Like satellite cells, laminin γ1 expression was unaffected in FAPs isolated from the PKO mice (Fig. 3i). Consistent with previous report, sorted PICs were PW1^+^Pax7^−^ (Fig. 3k). These cells also expressed PDGFRβ (Fig. 3k). In contrast to satellite cells and FAPs, laminin γ1 expression was absent in PICs isolated from the PKO mice (Fig. 3l). Altogether, our data suggest that laminin expression is abrogated in pericytes and PICs, but not satellite cells or FAPs in the PKO mice.

### Laminin inhibits proliferation of PDGFRβ^+^ cells

**Figure 4:**
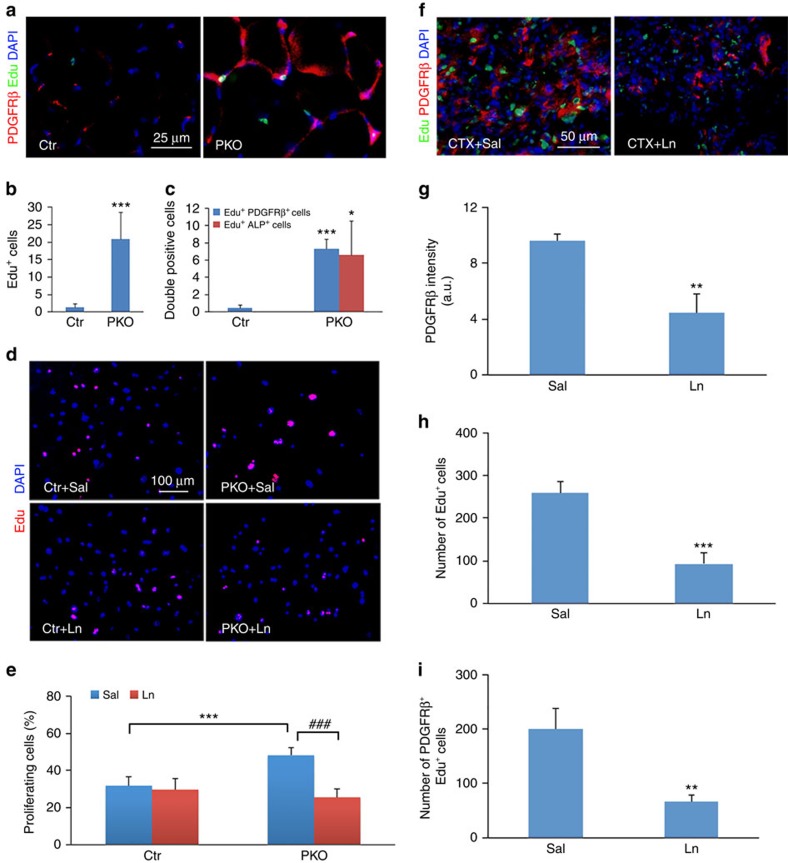
Laminin inhibits the proliferation of PDGFRβ^+^ cells. (**a**) Edu (green) incorporation and PDGFRβ (red) expression in hindlimb muscles from 2-month-old mice. (**b**,**c**) Quantification of total Edu^+^ cells (**b**) and Edu^+^PDGFRβ^+^ and Edu^+^ALP^+^ cells (**c**) in **a**; *n*=4. (**d**) Edu (red) incorporation in freshly isolated pericytes in the presence of saline or exogenous laminin. Ln, laminin; Sal, saline. (**e**) Quantification of Edu^+^ cells in **d**; *n*=6. (**f**) Edu (green) and PDGFRβ (red) expression 4 days after CTX injection in wild-type mice in the presence of saline or laminin. (**g**–**i**) Quantification of PDGFRβ expression (**g**), total Edu^+^ (**h**) and Edu^+^PDGFRβ^+^ cells (**i**) in tibialis anterior muscles; *n*=3. Scale bars, 25 μm in **a**, 100 μm in **d** and 50 μm in **f**. *P<0.05; **P<0.01; ***P<0.001; ^###^*P*<0.001 (Student's *t*-test). The results are shown as mean±s.d.

Enhanced PDGFRβ expression was observed in the PKO muscles (Fig. 2f,g). To investigate whether this is due to proliferation, we examined the incorporation of Edu, a BrdU analogue, *in vivo*. Compared with the control, 10-fold more Edu^+^ cells were found in the PKO muscles (Fig. 4a,b). Double immunostaining revealed significantly more proliferating pericytes (PDGFRβ^+^Edu^+^ and ALP^+^Edu^+^) in the PKO muscles (Fig. 4a,c), suggesting that loss of PDGFRβ^+^ cell-derived laminin leads to proliferation of these cells. Consistent with the time-course study, no differences in PDGFRβ expression and PDGFRβ^+^Ki67^+^ cell count were observed at P6 (Supplementary Fig. 5a-c). We next examined the proliferation of PDGFRβ^+^ cells *in vitro* and found that the PDGFRβ^+^ cells freshly isolated from PKO muscles incorporated significantly more Edu than those from control muscles (Fig. 4d,e). Interestingly, exogenous laminin (laminin-111) dramatically decreased Edu incorporation in the PDGFRβ^+^ cells isolated from the PKO but not control mice (Fig. 4d,e), suggesting that laminin negatively regulates the proliferation of PDGFRβ^+^ cells. In addition, although more caspase-3^+^ cells were found in the PKO muscles, no difference in the number of caspase-3^+^PDGFRβ^+^ cells was found between the control and PKO mice (Supplementary Fig. 6a). Consistent with these data, negligible number of TUNEL^+^ cells was observed in FACS-isolated control and PKO PDGFRβ^+^ cells (Supplementary Fig. 6b), suggesting that loss of laminin in the PDGFRβ^+^ cells does not induce their apoptosis.

To further investigate the role of laminin in the proliferation of the PDGFRβ^+^ cells in pathological conditions, we induced muscle injury using two well-characterized models: the cardiotoxin (CTX) model, which induces basement membrane degradation^4^,^44^, and the barium chloride model, which does not affect the basement membrane^16^,^45^. In the first model, the wild-type mice were injected with CTX in both tibialis anterior intramuscularly, followed by daily administration of saline and laminin to the left and right tibialis anterior, respectively. Four days after the injury, a large number of Edu^+^ and PDGFRβ^+^ cells were observed in saline-injected tibialis anterior (Fig. 4f). These cells, however, were dramatically reduced in laminin-injected tibialis anterior (Fig. 4f). Quantification revealed over 50% reduction in PDGFRβ intensity (Fig. 4g), total Edu^+^ cells (Fig. 4h) and PDGFRβ^+^Edu^+^ cells (Fig. 4i), suggesting that laminin inhibits proliferation of PDGFRβ^+^ cells after CTX-induced injury.

In the barium chloride model, the mice were treated similarly as described above. Consistent with previous reports^16^,^45^, laminin expression was unaffected after barium chloride injection (Supplementary Fig. 7a). PDGFRβ intensity and PDGFRβ^+^Edu^+^ number were extremely low (Supplementary Fig. 7b and d), suggesting that the PDGFRβ^+^ cells were not proliferating in this model. In addition, no significant differences in these parameters were observed between saline- and laminin-treated groups (Supplementary Fig. 7). These data again suggest that laminin inhibits the proliferation of PDGFRβ^+^ cells.

### Laminin promotes myogenesis of PDGFRβ^+^ cells

**Figure 5:**
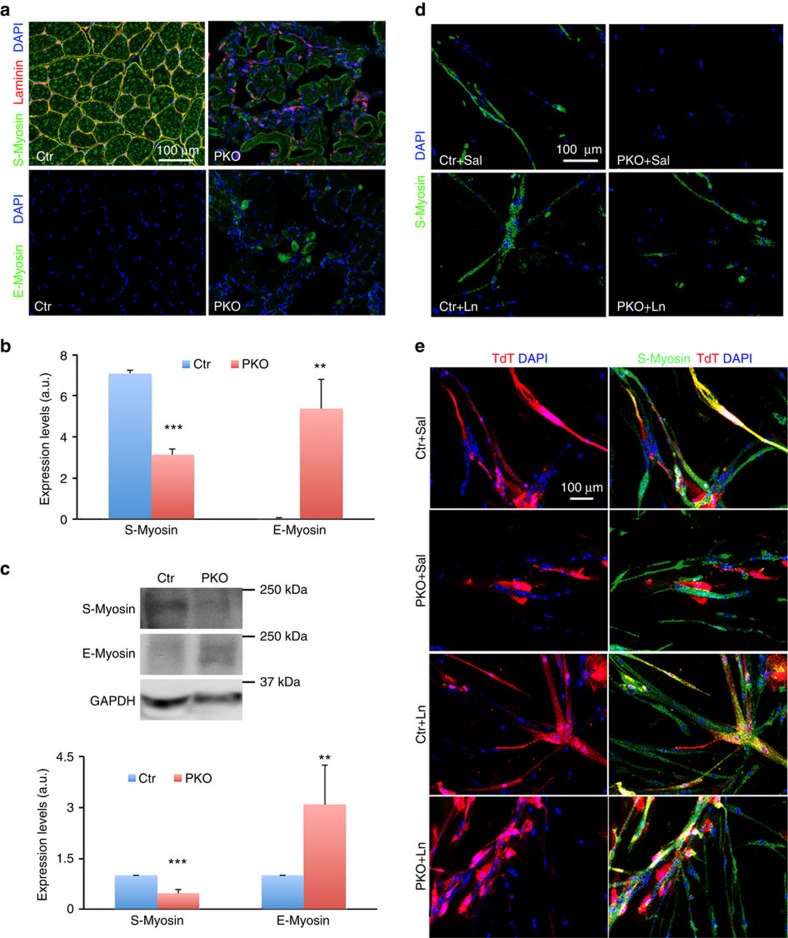
Laminin is indispensable for the myogenesis of PDGFRβ^+^ cells. (**a**) S-Myosin (green) and E-Myosin (green) expression in hindlimb muscles from 2-month-old mice. (**b**) Quantification of S-Myosin and E-myosin expression in **a**; *n*=3. (**c**) Western blot analysis of S-Myosin and E-Myosin expression in skeletal muscles. GAPDH was used as a loading control; *n*=6. (**d**) S-Myosin (green) expression in control and PKO PDGFRβ^+^ cells after 15 days in myogenic differentiation in the presence of saline or exogenous laminin. (**e**) Participation of TdT-labelled PDGFRβ^+^ cells (red) in the formation of S-Myosin (green)^+^ myofibres after co-culturing with wild-type satellite cells in myogenic medium. Scale bars, 100 μm in **a**,**d** and **e**. **P<0.01; ***P<0.001 (Student's *t*-test). The results are shown as mean±s.d.

Muscle development/maturation was examined using two markers: sarcomere myosin (S-Myosin), which is expressed in mature/adult muscles^2^,^46^, and embryonic myosin (E-Myosin), which is expressed in embryonic or injured muscles^4^,^47^. A high level of S-Myosin was distributed in a homogeneous pattern in hindlimb muscles of adult control mice (Fig. 5a). In age-matched PKO mice, however, S-Myosin was dramatically reduced (Fig. 5a). On the contrary, E-Myosin was absent in control muscles, but expressed at high levels in some small, central nucleated myofibres in the PKO mice (Fig. 5a). Quantification revealed that the changes in S-Myosin and E-Myosin were statistically significant (Fig. 5b). Similar results were obtained by western blots (Fig. 5c), suggesting that muscle development/maturation was compromised in the PKO mice. Consistent with the time-course study, neither S-Myosin nor E-Myosin expression was affected at P6 in the PKO mice (Supplementary Fig. 8).

During postnatal muscle development, myofibre size increases while myofibre number remains unchanged^48^,^49^,^50^. Since the PKO mice start to show MD phenotype at approximately P10, we hypothesize that PDGFRβ^+^ cell-derived laminin is indispensable for the growth of myofibres. To test this hypothesis, we grew freshly isolated PDGFRβ^+^ cells in myogenic medium. Fifteen days later, the control PDGFRβ^+^ cells formed S-Myosin-positive myotube-like structures (Fig. 5d). The PKO PDGFRβ^+^ cells, however, failed to express S-Myosin (Fig. 5d), suggesting laminin is essential for the myogenesis of PDGFRβ^+^ cells. Although exogenous laminin did not alter the control PDGFRβ^+^ cells, it did successfully induce S-Myosin^+^ myotube formation in PKO PDGFRβ^+^ cells (Fig. 5d). These data strongly suggest that myogenesis of PKO PDGFRβ^+^ cells can be induced by exogenous laminin.

Furthermore, we tested this hypothesis in a co-culture system. The control and PKO PDGFRβ^+^ cells permanently labelled with TdT were cultured with unlabelled wild-type satellite cells. The control PDGFRβ^+^ cells fused with satellite cells forming TdT^+^S-Myosin^+^ myofibres with or without exogenous laminin (Fig. 5e). The PKO PDGFRβ^+^ cells, on the other hand, failed to do so (Fig. 5e). In the presence of exogenous laminin, however, the PKO PDGFRβ^+^ cells were able to fuse with satellite cells and generate TdT^+^S-Myosin^+^ myofibres (Fig. 5e). Again, these data suggest that laminin is indispensable for the myogenesis of PDGFRβ^+^ cells.

### Laminin inhibits adipogenesis of PDGFRβ^+^ cells

**Figure 6:**
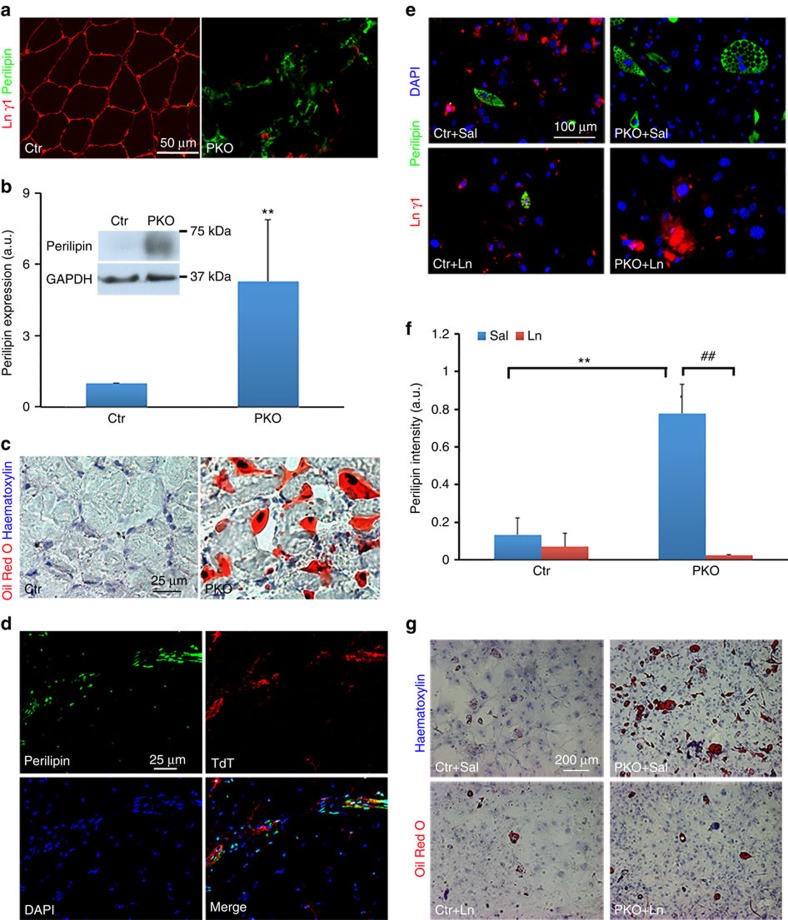
Laminin inhibits the adipogenesis of PDGFRβ^+^ cells. (**a**) Perilipin (green) and laminin γ1 (red) expression in hindlimb muscles from 2-month-old mice. (**b**) Western blot analysis of perilipin expression in skeletal muscles. GAPDH was used as a loading control; *n*=6. (**c**) Oil Red O staining of hindlimb muscles from 2-month-old mice. (**d**) Perilipin (green) expression co-localized with TdT (red) in the muscles of laminin-deficient reporter (F/F:Ai14:*Pdgfrβ-Cre*^+^) mice. (**e**) Perilipin (green) and laminin γ1 (red) expression in control and PKO PDGFRβ^+^ cells after 20 days in adipogenic differentiation in the presence of saline or exogenous laminin. (**f**) Quantification of perilipin expression in **e**; *n*=3. (**g**) Oil Red O and haematoxylin staining of primary PDGFRβ^+^ cells after adipogenic differentiation. Scale bars, 50 μm in **a**, 25 μm in **c** and **d**, 100 μm in **e** and 200 μm in **g**. **P<0.01; ^##^*P*<0.01 (Student's *t*-test). The results are shown as mean±s.d.

Adipocytes were found in the PKO but not control muscles (Fig. 1j), suggesting that loss of PDGFRβ^+^ cell-derived laminin may induce adipogenesis. Consistent with this observation, substantially higher levels of perilipin, an adipocyte marker, were found in hindlimb muscles from adult PKO mice (Fig. 6a,b). In addition, Oil Red O staining, which labels lipid droplets, demonstrated negligible and large number of adipocytes in the control and PKO muscles, respectively (Fig. 6c). These data suggest that PKO muscles contain significantly more adipocytes than the controls. Consistent with the time-course data, comparable levels of perilipin and retained Oil Red O were found in the control and PKO mice at P6 (Supplementary Fig. 9).

To determine the cellular origin of these adipocytes, we took advantage of the F/F:Ai14:*Pdgfrβ*^+^ mice, in which the PDGFRβ^+^ cells were permanently labelled with TdT. In these mice, perilipin expression partially co-localized with TdT (Fig. 6d), suggesting that the adipocytes are derived from both PDGFRβ^+^ and PDGFRβ^−^ cells. Next, we further tested this hypothesis *in vitro* using freshly isolated PDGFRβ^+^ cells. After 20 days in adipogenic medium, only limited numbers of control PDGFRβ^+^ cells expressed perilipin, whereas significantly more perilipin^+^ cells with typical adipocyte morphology (multiple intracellular vacuoles) were observed in the PKO PDGFRβ^+^ cells (Fig. 6e,f). Although exogenous laminin did not affect the control cells, it dramatically decreased the number of perilipin^+^ adipocytes in the PKO PDGFRβ^+^ cells (Fig. 6e,f). Oil Red O staining revealed similar results (Fig. 6g). These data strongly suggest that laminin inhibits adipogenesis of PDGFRβ^+^ cells.

### RNAseq analysis

**Figure 7:**
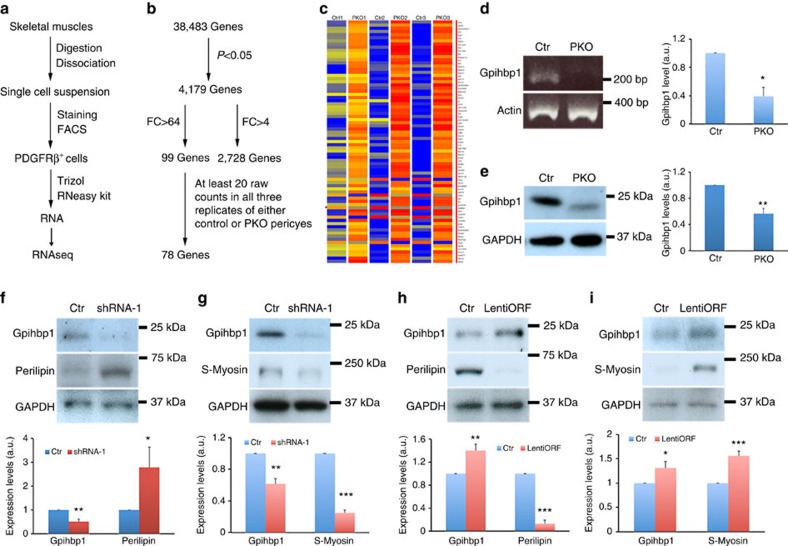
Laminin regulates the differentiation of PDGFRβ^+^ cells via gpihbp1. (**a**) Schematic illustration of sample preparation. (**b**) Illustration of RNAseq results. (**c**) Heatmap data for 78 genes identified. (**d**) PCR with reverse transcription analysis of *gpihbp1* expression in PDGFRβ^+^ cells. *Actin* was used as a loading control; *n*=3. (**e**) Western blot analysis of gpihbp1 expression in PDGFRβ^+^ cells. GAPDH was used as a loading control. *n*=6. (**f**,**g**) Western blot analysis of gpihbp1, perilipin and S-Myosin expression in wild-type PDGFRβ^+^ cells after gpihbp1 knockdown. GAPDH was used as a loading control; *n*=4. (**h**,**i**) Western blot analysis of gpihbp1, perilipin and S-Myosin expression in PKO PDGFRβ^+^ cells after gpihbp1 overexpression. GAPDH was used as a loading control; *n*=4. *P<0.05; **P<0.01; ***P<0.001 (Student's *t*-test). The results are shown as mean±s.d.

To explore the underlying molecular mechanisms, we performed RNAseq analysis using PDGFRβ^+^ cells freshly isolated from the control and PKO muscles (Fig. 7a). Owing to low density and strict gating, the PDGFRβ^+^ cells sorted from various experiments were pooled to generate one sample, and then three pairs of samples were used for RNAseq analysis. Among 38,483 genes identified, 4,179 showed statistical significance between the control and PKO PDGFRβ^+^ cells (Fig. 7b). Out of the 4,179 genes, 2,728 demonstrated a >4 fold change (FC) in expression between control and PKO PDGFRβ^+^ cells (Fig. 7b). To further narrow down the list, we set the FC cutoff to 64 (log FC>6) and identified 99 genes (Fig. 7b). In addition, at least 20 raw counts in all three replicates of either control or PKO samples were used to further filter this list, and we successfully identified 78 genes (Fig. 7c).

Among these genes, we focused on *Gpihbp1* (glycosylphosphatidylinositol anchored high density lipoprotein binding protein 1), given that: (i) it plays a critical role in lipid metabolism, and (ii) like PDGFRβ^+^ pericytes, it is exclusively found in capillaries^51^,^52^,^53^. We first validated its reduction in PKO pericytes at mRNA level (Fig. 7d). Similarly, western blots demonstrated significantly diminished expression of gpihbp1 in PKO pericytes (Fig. 7e), suggesting that loss of laminin decreases gpihbp1 expression in PDGFRβ^+^ cells at both mRNA and protein levels.

To investigate whether reduced gpihbp1 expression is responsible for loss of laminin-induced differentiation of PDGFRβ^+^ cells, we knocked down gpihbp1 expression in wild-type PDGFRβ^+^ cells by RNAi. Four lentiviral shRNA constructs were generated, and the most efficient construct was identified and used to decrease gpihbp1 expression in the wild-type PDGFRβ^+^ cells. By knockdown of gpihbp1 using this construct (Fig. 7f,g), we observed a significant increase in perilipin expression (Fig. 7f) and a significant reduction in S-Myosin expression (Fig. 7g), suggesting that gpihbp1 positively and negatively regulates myogenesis and adipogenesis of PDGFRβ^+^ cells, respectively. To determine whether increasing gpihbp1 in PKO PDGFRβ^+^ cells can reverse the observed molecular changes, we overexpressed gpihbp1 using a lentiviral construct. Compared with the control, this construct significantly upregulated gpihbp1 (Fig. 7h,i), decreased perilipin (Fig. 7h), and increased S-Myosin expression (Fig. 7i) in the PKO PDGFRβ^+^ cells, suggesting that increasing gpihbp1 can reverse adipogenesis and promote myogenesis of PDGFRβ^+^ cells. Altogether, our data suggest that laminin regulates the differentiation/fate determination of PDGFRβ^+^ cells via gpihbp1.

Furthermore, we also tested another significantly downregulated gene, mast cell chymase 1 (CMA1). Like gpihbp1, CMA1 expression was dramatically decreased at both mRNA and protein levels in the PKO PDGFRβ^+^ cells, compared with the controls (Supplementary Fig. 10a and b). Since CMA1 is predominantly found in extracellular space, CMA1 antibody was applied to the wild-type PDGFRβ^+^ cells to block CMA1 function. The wild-type cells treated with rabbit IgG were used as a control. Neither perilipin (Supplementary Fig. 10c) nor S-Myosin (Supplementary Fig. 10d) was affected by CMA1 antibody treatment. In addition, the PKO PDGFRβ^+^ cells were treated with recombinant CMA1 protein or BSA (control), and the same results were observed (Supplementary Fig. 10c and d), suggesting that laminin's effect on PDGFRβ^+^ cells is not mediated by CMA1.

### Laminin partially rescues MD in PKO mice

**Figure 8:**
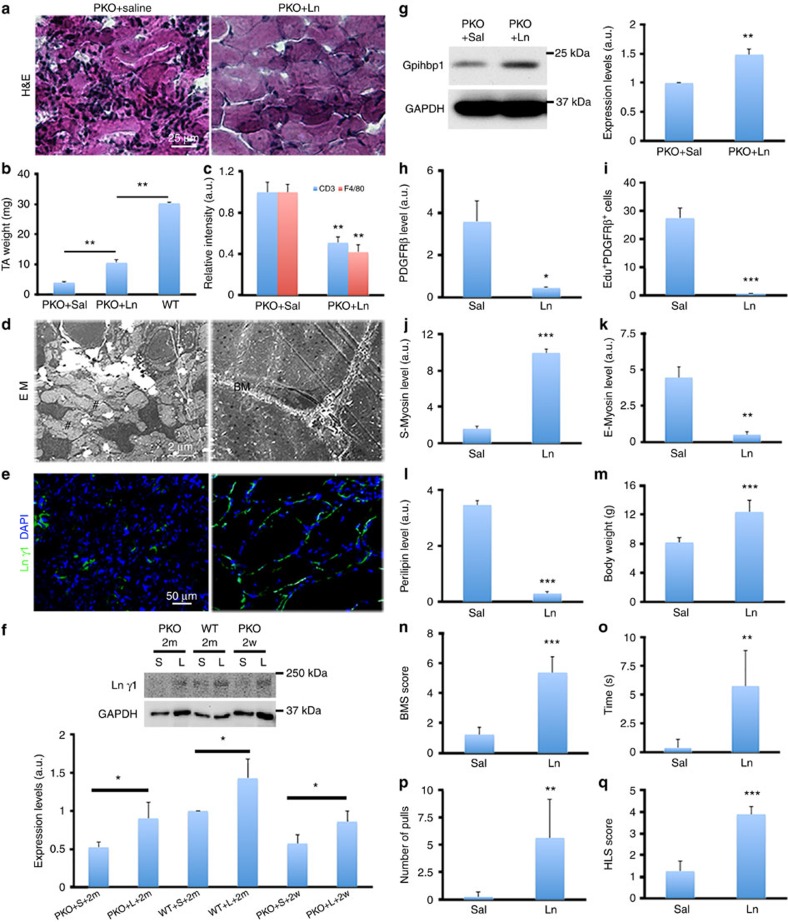
Exogenous laminin partially rescues MD phenotype in PKO mice. (**a**) H&E staining of hindlimb muscles from PKO mice after saline or laminin injection for 2 months. (**b**) Quantification of tibialis anterior muscle weight; *n*=4. (**c**) Quantification of CD3 and F4/80 expression in PKO muscles after saline (Sal) or laminin-111 (Ln) treatment for 2 months; *n*=3. (**d**) Representative ultrastructural images of PKO muscles after saline and laminin injection. BM, basement membrane; #, gap. (**e**) Laminin γ1 (green) expression in PKO muscles after saline or laminin injection for 2 months. (**f**) Western blot analysis of laminin γ1 expression in wild-type (WT) and PKO muscles after saline (S) or laminin-111 (L) treatment for 2 weeks (2w) or 2 months (2m); *n*=3. (**g**) Western blot analysis of gpihbp1 expression in PDGFRβ^+^ cells isolated from saline- or laminin-treated PKO mice; *n*=3. (**h**,**i**) Quantification of PDGFRβ expression (**h**) and PDGFRβ^+^Edu^+^ cells (**i**) in PKO mice after treatment; *n*=3. (**j**,**k**) Quantification of S-Myosin (**j**) and E-Myosin (**k**) expression in PKO mice after treatment; *n*=3. (**l**) Quantification of perilipin expression in PKO mice after treatment; *n*=3. (**m**–**q**) Quantification of body weight (**m**), BMS score (**n**), suspension time (**o**), number of pulls (**p**) and HLS score (**q**) in PKO mice treated bilaterally with saline or laminin for 2 months; *n*=8. Scale bars, 25 μm in **a**, 2 μm in **d** and 50 μm in **e**. *P<0.05; **P<0.01; ***P<0.001 (Student's *t*-test). The results are shown as mean±s.d.

To study the therapeutic effect of laminin in this model, we injected saline and exogenous laminin (laminin-111, 100 μg ml^−1^, 50 μl) into the left and right hindlimbs of the PKO mice, respectively. After 2 months, laminin-injected tibialis anterior muscle was significantly larger compared with saline-injected one, although they were still smaller than that from age-matched wild-type mice (Fig. 8b). Histology studies revealed dystrophic myofibres with infiltrated/accumulated cells in saline-injected muscles (Fig. 8a). Laminin injection reduced cell infiltration/accumulation and improved myofibre organization/structure (Fig. 8a). Immunohistochemical analyses revealed dramatically decreased infiltration of T cells and macrophages in the PKO muscles after laminin treatment (Fig. 8c), suggesting recovery. At the ultrastructural level, individual non-fused cells and gaps were observed in saline-injected muscles, while well-developed myofibres ensheathed by the basement membrane were found in laminin-injected muscles (Fig. 8d). These structural changes support improved myogenesis, suggesting that laminin is able to partially rescue the MD phenotype observed in PKO mice.

At the molecular level, laminin expression was dramatically increased around myofibres in laminin-injected muscles, compared to saline-injected ones (Fig. 8e), suggesting successful incorporation of laminin. Western blots confirmed enhanced laminin γ1 levels in the PKO mice 2 weeks and 2 months after laminin treatment (Fig. 8f). Next, gpihbp1 expression in the PDGFRβ^+^ cells from saline- or laminin-treated PKO mice were examined. Given that gpihbp1 is also expressed by capillary endothelial cells^51^,^52^, which outnumber PDGFRβ^+^ cells, freshly isolated PDGFRβ^+^ cells rather than muscle tissues were used for western blot analysis. A significantly higher level of gpihbp1 was found in PDGFRβ^+^ cells isolated from laminin-treated PKO mice, compared with those isolated from saline-treated PKO mice (Fig. 8g), suggesting that laminin treatment is able to rescue gpihbp1 expression in the PKO mice. In addition, we also examined gpihbp1 expression in dyW (*Lama2*-disrupted) mice, a widely used CMD model. Similarly, PDGFRβ^+^ cells from dyW muscles demonstrated reduced expression of gpihbp1 (Supplementary Fig. 11). More importantly, laminin treatment, which significantly reduces muscle pathology and improves muscle repair in these mice^26^,^27^, dramatically increased gpihbp1 expression in the PDGFRβ^+^ cells (Supplementary Fig. 11), again suggesting that the beneficial role of exogenous laminin in MD may be mediated by gpihbp1.

Next, PDGFRβ expression and PDGFRβ^+^Edu^+^ cells were substantially reduced in laminin-injected muscles compared with the controls (Fig. 8h,i), suggesting that intramuscularly injected laminin is able to inhibit the proliferation of PDGFRβ^+^ cells in the PKO mice. Furthermore, the injection of laminin significantly increased S-Myosin (Fig. 8j) and decreased E-Myosin (Fig. 8k) expression in the PKO mice, indicating improved myogenesis. In addition, perilipin expression was significantly diminished in laminin-injected hindlimbs (Fig. 8l). Consistent with these data, Oil Red O staining showed dramatically reduced number/size of lipid droplets in laminin-injected muscles, suggesting that exogenous laminin can reverse adipogenesis of PDGFRβ^+^ cells.

To evaluate the recovery of muscle function, laminin was injected daily into both hindlimbs of PKO mice for 2 months. Compared with saline-injected controls, laminin administration significantly increased animal body weight (Fig. 8m). In the Basso Mouse Scale for Locomotion (BMS) test^54^, laminin-injected mice demonstrated significantly higher BMS scores (Fig. 8n), indicating increased locomotion and better muscle function. Furthermore, in the hindlimb suspension test^55^, laminin injection substantially improved all three parameters (suspension time, number of pulls, and HLS score) in the PKO mice (Fig. 8o–q), indicating enhanced muscle strength. A representative video showing functional recovery of the hindlimbs is included (Supplementary Movie 1). Altogether, these behavioural data suggest that administration of exogenous laminin is able to partially increase muscle strength and improve muscle function in the PKO mice.

## Discussion

**Figure 9:**
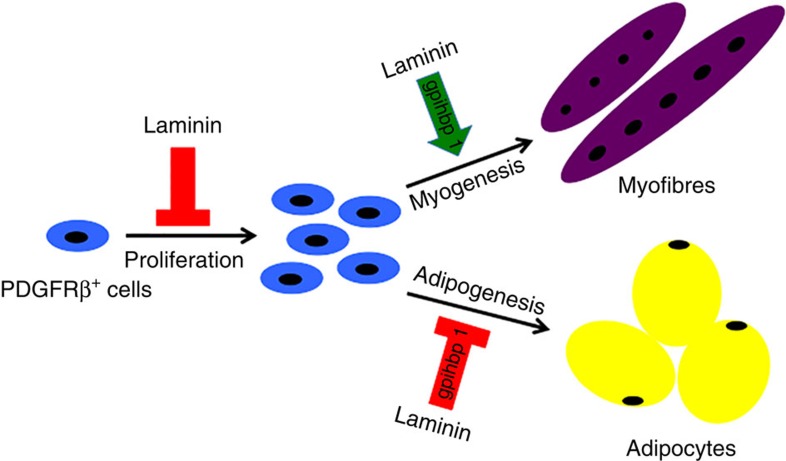
Schematic description of laminin's role in the stemness of PDGFRβ^+^ cells. Laminin negatively regulates the proliferation of PDGFRβ^+^ cells. In addition, laminin promotes myogenesis and inhibits adipogenesis of PDGFRβ^+^ cells via gpihbp1.

In this study, we show that PDGFRβ^+^ cell-derived laminin participates in muscle development/regeneration by negatively regulating the proliferation of PDGFRβ^+^ cells, inhibiting their adipogenesis and promoting their myogenesis. These findings are summarized in Fig. 9. The novelty and significance of this work are (i) we generated a new mouse line, which can be used in CMD research; (ii) we found that laminin actively regulates the stemness of PDGFRβ^+^ cells; (iii) we showed that exogenous laminin is able to partially rescue the muscle deficit in the PKO mice at the molecular, structural and functional levels; and (iv) we performed RNAseq analysis and identified gpihbp1 as a key regulator and molecular target for the differentiation of the PDGFRβ^+^ cells.

One of the most widely used CMD models is the dy^2J^ mice, which express near-normal amount of truncated protein due to a point mutation in the *Lama2* gene^22^,^23^,^24^. Although they replicate most of the pathological changes observed in human CMD patients, these dy^2J^ mice develop muscle symptoms at late (about 3.5 weeks after birth) rather than early postnatal stage^22^,^23^,^24^. A more aggressive CMD model is the *Lama2*-deficient mice^33^,^34^,^56^. These mice show muscle defects at early postnatal stage, but have a severe deficit in myelination^33^,^34^,^56^, which contributes to the MD phenotype and makes the interpretation of data difficult. In addition, most *Lama2*-deficient mice die within 2–4 weeks after birth^33^,^34^,^56^, preventing their use at a later stage. Like the *Lama2*-deficient mice, our PKO mice show muscle pathology in all voluntary muscles beginning at early postnatal stage (about P10) and progress rapidly, which closely replicates the time course observed in CMD patients. In addition, since only PDGFRβ^+^ cell-derived laminin is abrogated in the PKO mice, myelination defect is not detected in these mice. Furthermore, the PKO mice have a longer life span compared with the *Lama2*-deficient mice, enabling research at an older age possible. It should be noted, however, that (i) there are no known human CMD patients with *Lamc1* mutations and (ii) like the dy^2J^ and *Lama2*-deficient mice, the PKO mice are smaller in size, a phenotype not associated with human CMD pathology. Together, these data suggest that the PKO line may be used as an alternative, if not better, model in CMD research.

Lineage-tracing experiment and immunocytochemical analyses reveal that the PDGFRβ^+^ population we isolated contains pericytes and PICs, but not satellite cells or FAPs. In our model, wild-type PDGFRβ^+^ cells undergo myogenesis and contribute to muscle development/regeneration, suggesting that these cells, like satellite cells, have myogenic capability. These data are consistent with previous reports that transplantation of human pericytes into immune-deficient mdx mice generates myofibres expressing human dystrophy^13^, that pericytes participate in postnatal muscle growth/regeneration in both physiological and pathological conditions^12^, and that PICs are myogenic *in vitro* and contribute to muscle regeneration *in vivo*^17^,^18^,^19^,^20^. The next question then becomes: what are the differences between these cells and satellite cells in term of myogenesis? A time-course study demonstrated that satellite cells and pericytes have different kinetics in the expression of various myogenic transcription factors^13^. Another study elegantly showed that Pax7 is indispensable for the myogenic specification of PICs, whereas loss of Pax7 does not affect the proliferation and differentiation potential of satellite cells *in vitro*^17^. These studies strongly indicate that pericytes, PICs and satellite cells are three different populations with distinct myogenic kinetics and potentials. In addition, three labs independently report that muscle regeneration fails when Pax7^+^ satellite cells are genetically ablated^2^,^3^,^4^, suggesting that the myogenesis of pericytes and/or PICs is dependent on the presence of satellite cell population.

On loss of laminin, the PDGFRβ^+^ cells diminish their myogenic activity and obtain enhanced adipogenic activity. This observation is consistent with previous findings that both pericytes^16^,^21^ and PICs^17^,^20^ have adipogenic potential. We, for the first time, report that laminin regulates the differentiation and determines the fate of PDGFRβ^+^ cells (pericytes and PICs). In addition, collagen IV, another component of the basement membrane, has been shown to control the self-renewal of satellite cells and regulate muscle regeneration^40^. These data indicate that the basement membrane, which has direct contact with PDGFRβ^+^ cells and satellite cells, is more than just a supporting matrix. It more likely serves as an important niche to regulate the biological properties of these cells, probably via signalling the cells using its components. Further studies on how individual basement membrane components regulate the biological properties of these cells and how they interact with each other to form a cohesive/coordinate network will significantly enrich our knowledge on basement membrane biology and promote innovative therapies for many diseases, including MD.

Although laminin significantly improved muscle pathology/function, the PKO mice were still smaller and their muscles were weaker compared with age-matched controls. This partial recovery may be due to the local (only hindlimbs) intramuscular injection of laminin. In contrast to previous reports that systemic administration of laminin-111 reduced muscle pathology in *Lama2*-deficient mice^26^,^27^, we found intramuscular injection to be more efficient in the PKO mice. This discrepancy may be explained by the compromised vessel integrity in *Lama2*-deficient mice^57^, which may allow for the leakage of systemically delivered laminin to target organs. It should be noted, however, that intramuscular injection of laminin is impractical for human CMD patients owing to the large muscle size and limited diffusion of laminin. To find an alternative therapeutic option, we performed RNAseq analysis and identified gpihbp1 as a molecular target. Previous studies demonstrate that gpihbp1 is expressed in capillary endothelial cells and transports lipoprotein lipase from interstitial space to capillary lumen to regulate lipid metabolism^51^,^52^. It remains unclear, however, whether PDGFRβ^+^ pericytes, which cover endothelial cells in capillaries, are involved in this process. We, for the first time, report that PDGFRβ^+^ cells also express gpihbp1, suggesting that these cells may also regulate lipid metabolism and/or lipoprotein lipase transportation, possibly via gpihbp1. In addition, we show that gpihbp1 reversibly regulates the myogenesis and adipogenesis of PDGFRβ^+^ cells, indicating a critical role of gpihbp1 in PDGFRβ^+^ cell differentiation/fate determination. Targeting gpihbp1 may open new opportunities in developing effective therapies for CMD and other types of MD.

## Methods

### Animals

The *Pdgfrβ-Cre* transgenic mice were crossed with *Lamc1* floxed (F/F) mice to generate the PKO mice. For linear tracing experiment, Ai14 reporter line was crossed with the above-mentioned mice to generate control (Ai14:*Pdgfrβ-Cre*^+^) and laminin-deficient (F/F:Ai14:*Pdgfrβ-Cre*^+^) reporter lines. The dyW mice were obtained by crossing *Lama2*^+/−^ mice (The Jackson Lab). The PKO mice used in this study were in a mixed background. The mice (both genders) at various ages were used (see the ‘Results' section for details). All the mice were maintained in the Comparative Biosciences Center at The Rockefeller University and animal facility at the University of Minnesota with free access to water and food. The experimental procedures were in accordance with the NIH guide for the care and use of animals and were approved by the Institutional Animal Care and Use Committee at The Rockefeller University and University of Minnesota.

### Cell culture

The primary PDGFRβ^+^ cells were grown in pericyte medium (ScienCell, 1201), myogenic differentiation medium (DMEM with 2% horse serum and 1% penicillin/streptomycin) or adipogenic differentiation medium (MesenCult Basal Medium supplemented with Adipogenic Stimulatory Supplement, STEMCELL, 05501 and 05503) at 37 °C with 5% CO_2_.

The satellite cells were grown in satellite cell medium (DMEM with 10% FBS, 10% horse serum and 1% penicillin/streptomycin) at 37 °C with 5% CO_2_ for 16 h.

### Muscle dissection and preparation

The mice were anaesthetized and hindlimb muscles were carefully dissected and minced with scissors and blades. The minced muscle fragments were incubated with 0.2% (w/v) type-2 collagenase (Worthington, LS004176) in DMEM at 37 °C for 2 h, followed by 3 × 15 min incubations with 0.25% trypsin/EDTA at 37 °C. After trituration and centrifugation at 700*g* for 6 min, the supernatant was discarded and the pellet resuspended in RBC lysis buffer. After centrifugation, the pellet was resuspended in DMEM+10% FBS and passed through a 40-μm cell strainer to remove aggregates. The resulting single cell solution was centrifuged again at 700*g* for 6 min and resuspended in sorting buffer (20 mM HEPES pH 7.0, 1 mM EDTA, 1% BSA in 1 × Ca/Mg^2+^-free phosphate-buffered saline (PBS) pH 7.0). The cells were then stained with different antibodies and subjected to FACS sorting.

### Cell sorting

The PDGFRβ^+^ cells were isolated by FACS as described previously^12^,^13^,^16^. Briefly, the single cell solution was stained with PDGFRβ-PE (eBioscience, 12-1402, 1:100), CD45-FITC (eBioscience, 11-0451, 1:100) and CD31-FITC (eBioscience, 11-0311, 1:100) on ice for 30 min. After extensive washing, DAPI was added and PE^+^FITC^−^ cells were sorted using the BD FACS Aria-II sorter. For co-culture experiment, TdT^+^FITC^−^ cells were sorted similarly from control and laminin-deficient reporter mice. The satellite cells were isolated as described previously^39^,^40^. Briefly, the single cell solution was stained with Biotin-SM/C-2.6 (a generous gift from Dr So-ichiro Fukada, Osaka University, Japan, 1:100), CD45-FITC (eBioscience, 11-0451, 1:100), CD31-FITC (eBioscience, 11-0311, 1:100) and Sca1-FITC (eBioscience, 11-5981, 1:100) for 30 min on ice, followed by Streptavidin-APC (eBioscience, 17-4317, 1:100) on ice for 30 min. After extensive washing, DAPI was added and APC^+^FITC^−^ cells were sorted using the BD FACS Aria-II sorter. FAPs were isolated as described previously^41^. Briefly, the single cell solution was stained with PDGFRα-APC (eBioscience, 17-1401, 1:100), CD45-FITC (eBioscience, 11-0451, 1:100) and CD31-FITC (eBioscience, 11-0311, 1:100) on ice for 30 min. After extensive washing, DAPI was added and the APC^+^FITC^−^ cells were sorted using the Sony SH800 sorter. PICs were isolated as described previously^17^. Briefly, the single cell solution was stained with CD34-biotin (eBioscience, 13-0341, 1:100), Sca1-PE (eBioscience, 12-5981, 1:100) and CD45-FITC (eBioscience, 11-0451, 1:100) on ice for 30 min, followed by Streptavidin-APC (eBioscience, 17-4317, 1:100) on ice for 30 min. After extensive washing, DAPI was added and APC^+^PE^Med^ FITC^−^ cells were sorted using the Sony SH800 sorter. Forward and side light scatters and DAPI were used to set a primary gate to exclude dead cells and small debris. Fluorescence minus one control for PE, APC and FITC were used to show individual gating boundaries. The cells were sorted either in culturing medium for *in vitro* experiments or in Trizol reagent (Life Technologies, 15596026) for RNAseq analysis.

### Proliferation assays

Proliferation was examined using the Click-iT Plus EdU Alexa Fluor 594 Imaging Kit (Life Technologies, C10639). For *in vitro* studies, freshly isolated control and PKO PDGFRβ^+^ cells were plated in 24-well plates in pericyte medium and treated with saline or laminin-111 (Invitrogen, 23017-015, 5 μg ml^−1^) for 3 days. Edu (8 μM) was added to the cells for 24 h, followed by fixation and immunodetection, according to the manufacturer's instructions. For *in vivo* studies, the adult control and PKO mice were injected with Edu (0.1 mg ml^−1^, 100 μl) intraperitoneally and killed 24 h later. Proliferation in control and PKO mice at postnatal day 6 was examined by Ki67 immunostaining.

### *In vitro* myogenic differentiation assays

In a simple myogenic differentiation assay involving only PDGFRβ^+^ cells, sorted control and PKO PDGFRβ^+^ cells were grown in 24-well plates in pericyte medium. When they reached 70% confluence, the pericyte medium was replaced with myogenic differentiation medium. After 15 days, the cells were fixed in 4% PFA and immunostained with anti-S-Myosin (DSHB, MF20, 1:100) antibody.

In a more complicated myogenic differentiation assay involving PDGFRβ^+^ cells and satellite cells, we utilized a widely used co-culture protocol with minor modifications^58^. Briefly, the control and PKO PDGFRβ^+^ cells permanently labelled with TdT were cultured with freshly sorted wild-type satellite cells in growth medium (50% satellite cell medium+50% pericyte medium) for 4 days. On day 5, the growth medium was replaced with myogenic differentiation medium supplemented with saline or laminin (Invitrogen, 23017-015, 5 μg ml^−1^). After 8 days, the cells were fixed in 4% PFA and stained with anti-S-Myosin antibody (DSHB, MF20, 1:100). The expression of TdT and S-Myosin was analysed under Zeiss LSM 710 confocal microscope.

### *In vitro* adipogenic differentiation assay

Adipogenic differentiation was induced using adipogenic differentiation medium. Briefly, freshly isolated control and PKO PDGFRβ^+^ cells were plated in 24-well plates in pericyte medium. When they reached 60% confluence, pericyte medium was replaced with adipogenic differentiation medium containing saline or laminin-111 (Invitrogen, 23017-015, 5 μg ml^−1^). After 20 days, the cells were fixed and subjected to perilipin immunostaining and Oil Red O staining.

### RNAseq analysis

Muscle PDGFRβ^+^ cells from 15 to 20 mice (2–4 months old) were sorted into Trizol reagent and stored at −80 °C. The sorted cells from three independent sorting experiments (45–60 mice) were pooled into one sample and subjected to RNA isolation through phenol extraction, followed by a purification step using the RNeasy Plus Mini Kit (Qiagen, 74134) according to the manufacturer's instructions. Three control and three PKO (pooled) samples were used for RNAseq analysis. The quality of RNA was examined by running samples on an Agilent 2100 Bioanalyzer, and only those with RNA Integrality Number (RIN) higher than 8.0 underwent RNAseq analysis. Owing to low RNA levels, the samples were amplified with the SMARTer Ultra Low Input RNA complementary DNA (cDNA) preparation kit (Clontech). After amplification, the cDNA samples were fragmented and sequencing libraries were prepared. Single-end 50 bp sequencing was then performed on a HiSeq 2,500 sequencer (Illumina). The RNAseq data were deposited in NCBI Sequence Read Archive (SRA Accession: SRP071344) and analysed using the StrandNGS platform (StrandNGS) according to the manufacturer's instructions.

### PCR with reverse transcription

The muscle PDGFRβ^+^ cells isolated from the wild-type and PKO mice were subjected to RNA extraction. Three pairs of independent RNA samples (each sample contains RNA from 45 to 60 mice) were subjected to PCR with reverse transcription analysis. Briefly, equal amounts of purified RNA were reverse-transcribed to cDNA using the SuperScript III First-Strand Synthesis System (Invitrogen, 18080-051) as instructed. The expression levels of *Gpihbp1, Cma1* and *Actin* were examined by PCR using the following primers. *Gpihbp1*: 5′- AGCAAACCCTTCTGCATCAC -3′, 5′- ACCCAGAGGGTTCTGGACTT -3′; *Cma1*: 5′- GAGAACTACCTGTCGGCCTG -3′, 5′- GTTCCCACACCTAGGGTTAGC -3′; *Actin*: 5′- ACAGCTGAGAGGGAAATCGT -3′, 5′- TGCTAGGAGCCAGAGCAGTA -3′. The sizes of PCR products for *Gpihbp1*, *Cma1* and *Actin* are 201 bp, 251 bp and 364 bp, respectively. The expression levels of *gpihbp1* and *cma1* in each sample were normalized to the housekeeping gene *Actin*.

### Lentivirus generation

For RNAi, four mouse gpihbp1 shRNA lentiviral constructs (Clone IDs: TRCN0000178358, TRCN0000178162, TRCN0000182215 and TRCN0000177537) were purchased from TRC Lentiviral shRNA. A control construct was purchased from Addgene. These lentivectors and lentiviral packaging plasmids (psPAX2 and pMD2.G from Addgene) were co-transfected into HEK 293T cells (ATCC, CRL-3216) using Lipofectamine 2000 (Life Technologies).

For over-expression, gpihbp1 cloned into the pLenti-GIII-EF1α vector (LV172809) and the empty vector (LV588) were purchased from Applied Biological Materials. The empty vector and gpihbp1-overexpressing construct were co-transfected into HEK293T cells with lentiviral packaging plasmids using Lipofectamine 2000. After 3.5 days, the media containing packaged lentiviruses were collected and filtered through 0.45 μm filters. The lentiviruses were further concentrated by ultracentrifuge at 110,000*g* and 4 °C for 2 h.

### Gpihbp1 knockdown or overexpression

The control and PKO pericytes were used for knockdown and overexpression experiments, respectively. Briefly, the pericytes were plated in 12-well plates. When they reached 60–70% confluence, 8 μg per ml polybrene and appropriate amount of lentivirus were added to reach a final MOI of 10 (10 lentiviruses per cell). The medium was replaced 16 h after transduction and every 2 days thereafter. By day 10, the pericyte lysates were collected.

### Animal treatments

For laminin injection, saline (50 μl) and laminin-111 (100 μg ml^−1^, 50 μl) were injected into the shaved left and right hindlimbs, respectively, of PKO mice once a day for 14 or 60 days. Edu (0.1 mg ml^−1^, 100 μl) was injected intraperitoneally 24 h before these mice were killed.

For muscle injury, the wild-type mice were anaesthetized as described before and shaved on the hindlimbs. Cardiotoxin (10 μM, 50 μl) or barium chloride (1%, 100 μl) was injected intramuscularly into the tibialis anterior of both hindlimbs. Saline (50 μl) and laminin-111 (100 μg ml^−1^, 50 μl) were injected intramuscularly into the left and right tibialis anterior of these mice, respectively, once a day until killed. Edu (0.1 mg ml^−1^, 100 μl) was injected intraperitoneally 24 h before being killed.

### Hindlimb suspension test

To evaluate the hindlimb muscle strength, weakness and fatigue, the hindlimb suspension test was performed^55^. Briefly, a 50 ml conical tube containing cotton balls at the bottom was placed vertically. Each mouse was placed face down inside the tube with its hind legs over the rim of the tube. Three parameters were recorded: the time the mouse spent hanging off the rim, the number of pulls (attempts to lift its body using its hindlimb muscles) it tried while suspended, and the hindlimb suspension (HLS) score measured according to the posture of its hind legs and tail (on a scale of 0–4 with 0 being the weakest). If the mouse fell instantly (could not suspend from the tube), default scores 0 (hang time), 0 (pulls), 1 (HLS) were assigned.

### BMS test

The hindlimb functional deficits were assessed using the BMS test, a rating system widely used to examine the locomotor recovery^54^. Briefly, the mice were placed in an open field, and their ankle movement, plantar placement, stepping, coordination, paw position, trunk instability and tail position were scored using the BMS system. The scores range from 0 to 9, with 0 being complete paralysis and 9 being normal movement of the hindlimbs.

### Immunohistochemistry and immunocytochemistry

Ten micrometre-thick transverse muscle sections were obtained using a cryostat. The muscle sections or sorted cells were immunostained with anti-laminin γ1 (Abcam, AB3297, 1:200; NeoMarkers, RT-795-P0, 1:100), anti-laminin-111 (Sigma, L9393, 1:1,000), anti-CD3 (eBioscience, 14-0032, 1:100), anti-F4/80-Biotin (eBioscience, 13-4801, 1:100), anti-PDGFRα (eBioscience, 14-1401, 1:100), anti-PEG3/PW1 (Bioss, bs-1870R, 1:100), anti-cleaved caspase-3 (Cell Signaling, 9661, 1:200), anti-CD13-FITC (BD Pharmingen, 558744, 1:200), anti-CD31 (BD Pharmingen, 553370, 1:200), anti-CD31-FITC (BD Pharmingen, 553372, 1:200), anti-CD45-FITC (eBioscience, 11-0451, 1:200), anti-SMA-FITC (Sigma, F3777, 1:1,000), anti-SM/C-2.6 (generous gift from Dr Fukada), anti-Pax7 (DSHB, Pax7, 1:100), anti-ALP (R&D, AF2910, 1:200), anti-fibroblast (Acris Antibodies, BM4018, 1:500), anti-PDGFRβ (eBioscience, 14-1402, 1:100), anti-S-Myosin (DSHB, MF-20, 1:100), anti-E-Myosin (DSHB, GTX113561, 1:10), anti-perilipin (Sigma, P1998, 1:500) or anti-Ki67 (Millipore, AB9260, 1:1,000) antibodies overnight at 4 °C, followed by fluorescent secondary antibodies (Invitrogen) for 1 h at room temperature. After mounting, the sections were examined and photographed with an Axiovert 200 (Zeiss) microscopy or LSM 710 confocal microscopy. Sections from the one-third and two-third of the muscles along the anterior/posterior axis were used for quantification. At least three random images from each section were used.

### Histology

H&E staining and Oil Red O staining were performed according to standard protocols. For H&E staining, the muscle sections were fixed in 4% PFA and incubated in haematoxylin (Fisher Scientific, SH30-500D) for 15 min. After washing, the sections were differentiated in 1% acid alcohol and saturated lithium carbonate solution, followed by five dips in eosin (Sigma, HT-110132) and extensive washing. Next, the sections were dehydrated in gradient alcohol and xylene and mounted in DPX. For Oil Red O staining, the sections were fixed in PFA, washed in PBS and rinsed in 60% isopropanol. After incubating in Oil Red O (Sigma, O1391) working solution for 15 min, the sections were rinsed in 60% isopropanol and stained in haematoxylin for 2 min. Then, the sections were washed in water and mounted in VECTASHIELD mounting medium (Vector Lab, H-1,000).

### Western blot

Hindlimb skeletal muscles were homogenized in tissue lysis buffer (100 mM Tris, pH 8, 1% SDS, 200 mM NaCl, 5 mM EDTA, 1X protease inhibitor cocktail, 1X phosphatase inhibitor cocktail). The pericytes were collected and lysed with RIPA buffer (50 mM Tris, pH 7.4, 1% NP-40, 0.5% Na-deoxycholate, 1% SDS, 150 mM NaCl, 2 mM EDTA, 1X protease inhibitor cocktail and 1X phosphatase inhibitor cocktail). Total protein concentrations were determined using the Bio-Rad protein assay kit. Equal amounts of proteins were loaded and separated on 10% SDS–PAGE or 4–20%Tris-Glycine Mini Protein Gels (Life Technologies) and then transferred to polyvinylidene fluoride membranes (Millipore). The membranes were then incubated with anti-laminin γ1 (NeoMarkers, RT-795-P0, 1:100), anti-PDGFRβ (Cell Signaling, 3169S, 1:500), anti-S-Myosin (DSHB, MF-20, 1:100), anti-E-Myosin (DSHB, GTX113561, 1:10), anti-perilipin (Sigma, P1998, 1:500), anti-gpihbp1 (Thermo Scientific, PA1-16976, 1:200) or anti-GAPDH (Abcam, ab9484, 1:1,000) antibodies at 4 °C overnight, followed by incubation with HRP-conjugated secondary antibodies (Jackson ImmunoResearch Lab). The proteins were visualized by SuperSignal West Pico Chemiluminescent Substrate (Pierce). The density of bands was normalized to GAPDH and quantified using NIH Image J. The full-size images for all the blots are included in Supplementary Fig. 12.

### TUNEL assay

Cell death was examined using the *In Situ* Cell Death Detection Kit, TMR red (Roche), according to the manufacturer's protocol. Briefly, the sorted pericytes or muscle sections were fixed in 4% PFA and permeabilized on ice, followed by incubation with TUNEL reaction mixture for 60 min at 37 °C. After extensive washing, the sections were examined under fluorescence microscopy.

### Electron microscopy analysis

Two-month-old mice were anaesthetized and perfused with PBS followed by 0.1 M sodium cacodylate buffer containing 2% paraformaldehyde and 2% glutaraldehyde. After perfusion, the skeletal muscles from the hindlimb were dissected out and fixed overnight. After post-fixing in 1% osmium tetroxide and 1% K-ferrocyanide, the tissue was *en bloc* stained with 2% uranyl acetate and embedded in resin. Ultra-thin sections were cut on a Reichert-Jung Ultracut E microtome and post-stained with 2% uranyl acetate and 1% lead citrate. The sections were examined and photographed using JEOL100CXII at 80 KV.

### Statistics

The results are shown as mean±s.d. Student's *t*-test, performed by SPSS Statistics, was used to analyse differences between the two groups. The size of samples was chosen to allow a statistical analysis of the results. Sample number (*n*) represents biological replicates. All the experiments were replicated at least twice.

## Additional information

**Accession codes:** The RNAseq data are deposited in NCBI Sequence Read Archive under the accession number: SRP071344.

**How to cite this article:** Yao, Y. *et al*. Laminin regulates PDGFRβ^+^ cell stemness and muscle development. *Nat. Commun.* 7:11415 doi: 10.1038/ncomms11415 (2016).

## Supplementary Material

Supplementary Figures Supplementary Figures 1-12

Supplementary Movie 1 Laminin injection partially improves hind-limb function in PKO mice. PKO mice were intramuscularly injected with laminin-111 (100 μg ml-1, 50 μl) or saline in both hind-limbs for 2 months. Compared to saline-injected mice, laminin-111-injected ones demonstrated increased locomotion and substantially improved muscle function. This movie shows one representative mouse (out of 8) from each group. Left: Laminin-111 injection; Right: Saline injection.

## References

[b1] McPhee S. J. Pathophysiology of Disease: an Introduction to Clinical Medicine Prentice-Hall International (1995).

[b2] Lepper C., Partridge T. A. & Fan C. M. An absolute requirement for Pax7-positive satellite cells in acute injury-induced skeletal muscle regeneration. Development 138, 3639–3646 (2011). 21828092 10.1242/dev.067595 PMC3152922

[b3] Sambasivan R. . Pax7-expressing satellite cells are indispensable for adult skeletal muscle regeneration. Development 138, 3647–3656 (2011). 21828093 10.1242/dev.067587

[b4] Murphy M. M., Lawson J. A., Mathew S. J., Hutcheson D. A. & Kardon G. Satellite cells, connective tissue fibroblasts and their interactions are crucial for muscle regeneration. Development 138, 3625–3637 (2011). 21828091 10.1242/dev.064162 PMC3152921

[b5] Kuang S., Charge S. B., Seale P., Huh M. & Rudnicki M. A. Distinct roles for Pax7 and Pax3 in adult regenerative myogenesis. J. Cell Biol. 172, 103–113 (2006). 16391000 10.1083/jcb.200508001 PMC2063538

[b6] Relaix F. & Zammit P. S. Satellite cells are essential for skeletal muscle regeneration: the cell on the edge returns centre stage. Development 139, 2845–2856 (2012). 22833472 10.1242/dev.069088

[b7] von Maltzahn J., Jones A. E., Parks R. J. & Rudnicki M. A. Pax7 is critical for the normal function of satellite cells in adult skeletal muscle. Proc. Natl Acad. Sci. USA 110, 16474–16479 (2013). 24065826 10.1073/pnas.1307680110 PMC3799311

[b8] Morgan J. E., Pagel C. N., Sherratt T. & Partridge T. A. Long-term persistence and migration of myogenic cells injected into pre-irradiated muscles of mdx mice. J. Neurol. Sci. 115, 191–200 (1993). 7683332 10.1016/0022-510x(93)90224-m

[b9] Beauchamp J. R., Morgan J. E., Pagel C. N. & Partridge T. A. Dynamics of myoblast transplantation reveal a discrete minority of precursors with stem cell-like properties as the myogenic source. J. Cell Biol. 144, 1113–1122 (1999). 10087257 10.1083/jcb.144.6.1113 PMC2150577

[b10] Partridge T. A. Invited review: myoblast transfer: a possible therapy for inherited myopathies? Muscle Nerve 14, 197–212 (1991). 2041542 10.1002/mus.880140302

[b11] Montarras D. . Direct isolation of satellite cells for skeletal muscle regeneration. Science 309, 2064–2067 (2005). 16141372 10.1126/science.1114758

[b12] Dellavalle A. . Pericytes resident in postnatal skeletal muscle differentiate into muscle fibres and generate satellite cells. Nat. Commun. 2, 499 (2011). 21988915 10.1038/ncomms1508

[b13] Dellavalle A. . Pericytes of human skeletal muscle are myogenic precursors distinct from satellite cells. Nat. Cell Biol. 9, 255–267 (2007). 17293855 10.1038/ncb1542

[b14] Sampaolesi M. . Mesoangioblast stem cells ameliorate muscle function in dystrophic dogs. Nature 444, 574–579 (2006). 17108972 10.1038/nature05282

[b15] Sampaolesi M. . Cell therapy of alpha-sarcoglycan null dystrophic mice through intra-arterial delivery of mesoangioblasts. Science 301, 487–492 (2003). 12855815 10.1126/science.1082254

[b16] Birbrair A. . Role of pericytes in skeletal muscle regeneration and fat accumulation. Stem Cells Dev. 22, 2298–2314 (2013). 23517218 10.1089/scd.2012.0647 PMC3730538

[b17] Mitchell K. J. . Identification and characterization of a non-satellite cell muscle resident progenitor during postnatal development. Nat. Cell Biol. 12, 257–266 (2010). 20118923 10.1038/ncb2025

[b18] Relaix F. . Pw1, a novel zinc finger gene implicated in the myogenic and neuronal lineages. Dev. Biol. 177, 383–396 (1996). 8806818 10.1006/dbio.1996.0172

[b19] Schwarzkopf M., Coletti D., Sassoon D. & Marazzi G. Muscle cachexia is regulated by a p53-PW1/Peg3-dependent pathway. Genes Dev. 20, 3440–3452 (2006). 17182869 10.1101/gad.412606 PMC1698450

[b20] Pannerec A., Formicola L., Besson V., Marazzi G. & Sassoon D. A. Defining skeletal muscle resident progenitors and their cell fate potentials. Development 140, 2879–2891 (2013). 23739133 10.1242/dev.089326

[b21] Armulik A., Genove G. & Betsholtz C. Pericytes: developmental, physiological, and pathological perspectives, problems, and promises. Dev. Cell 21, 193–215 (2011). 21839917 10.1016/j.devcel.2011.07.001

[b22] Meier H. & Southard J. L. Muscular dystrophy in the mouse caused by an allele at the dy-locus. Life Sci. 9, 137–144 (1970). 5434356 10.1016/0024-3205(70)90306-1

[b23] Sunada Y., Bernier S. M., Utani A., Yamada Y. & Campbell K. P. Identification of a novel mutant transcript of laminin alpha 2 chain gene responsible for muscular dystrophy and dysmyelination in dy2J mice. Hum. Mol. Genet. 4, 1055–1061 (1995). 7655459 10.1093/hmg/4.6.1055

[b24] Xu H., Wu X. R., Wewer U. M. & Engvall E. Murine muscular dystrophy caused by a mutation in the laminin alpha 2 (Lama2) gene. Nat. Genet. 8, 297–302 (1994). 7874173 10.1038/ng1194-297

[b25] Durbeej M. & Campbell K. P. Muscular dystrophies involving the dystrophin-glycoprotein complex: an overview of current mouse models. Curr. Opin. Genet. Dev. 12, 349–361 (2002). 12076680 10.1016/s0959-437x(02)00309-x

[b26] Rooney J. E., Knapp J. R., Hodges B. L., Wuebbles R. D. & Burkin D. J. Laminin-111 protein therapy reduces muscle pathology and improves viability of a mouse model of merosin-deficient congenital muscular dystrophy. Am. J. Pathol. 180, 1593–1602 (2012). 22322301 10.1016/j.ajpath.2011.12.019 PMC3349899

[b27] Van Ry P. M., Minogue P., Hodges B. L. & Burkin D. J. Laminin-111 improves muscle repair in a mouse model of merosin-deficient congenital muscular dystrophy. Hum. Mol. Genet. 23, 383–396 (2014). 24009313 10.1093/hmg/ddt428 PMC3869356

[b28] Goudenege S. . Laminin-111: a potential therapeutic agent for Duchenne muscular dystrophy. Mol. Ther. 18, 2155–2163 (2010). 20683444 10.1038/mt.2010.165 PMC2997583

[b29] Rooney J. E., Gurpur P. B. & Burkin D. J. Laminin-111 protein therapy prevents muscle disease in the mdx mouse model for Duchenne muscular dystrophy. Proc. Natl Acad. Sci. USA 106, 7991–7996 (2009). 19416897 10.1073/pnas.0811599106 PMC2683113

[b30] Yao Y., Chen Z. L., Norris E. H. & Strickland S. Astrocytic laminin regulates pericyte differentiation and maintains blood brain barrier integrity. Nat. Commun. 5, 3413 (2014). 24583950 10.1038/ncomms4413 PMC3992931

[b31] Yao Y., Norris E. H. & Strickland S. The cellular origin of laminin determines its role in blood pressure regulation. Cell. Mol. Life Sci. 72, 999–1008 (2015). 25216704 10.1007/s00018-014-1732-y PMC4323860

[b32] Chen Z. L. & Strickland S. Laminin gamma1 is critical for Schwann cell differentiation, axon myelination, and regeneration in the peripheral nerve. J. Cell Biol. 163, 889–899 (2003). 14638863 10.1083/jcb.200307068 PMC2173689

[b33] Miyagoe Y. . Laminin alpha2 chain-null mutant mice by targeted disruption of the Lama2 gene: a new model of merosin (laminin 2)-deficient congenital muscular dystrophy. FEBS Lett. 415, 33–39 (1997). 9326364 10.1016/s0014-5793(97)01007-7

[b34] Kuang W. . Merosin-deficient congenital muscular dystrophy. Partial genetic correction in two mouse models. J. Clin. Invest. 102, 844–852 (1998). 9710454 10.1172/JCI3705 PMC508948

[b35] Doe J. A. . Transgenic overexpression of the alpha7 integrin reduces muscle pathology and improves viability in the dy(W) mouse model of merosin-deficient congenital muscular dystrophy type 1A. J. Cell Sci. 124, 2287–2297 (2011). 21652631 10.1242/jcs.083311 PMC3113674

[b36] Girgenrath M., Beermann M. L., Vishnudas V. K., Homma S. & Miller J. B. Pathology is alleviated by doxycycline in a laminin-alpha2-null model of congenital muscular dystrophy. Ann. Neurol. 65, 47–56 (2009). 19086074 10.1002/ana.21523 PMC2639627

[b37] Meinen S., Barzaghi P., Lin S., Lochmuller H. & Ruegg M. A. Linker molecules between laminins and dystroglycan ameliorate laminin-alpha2-deficient muscular dystrophy at all disease stages. J. Cell Biol. 176, 979–993 (2007). 17389231 10.1083/jcb.200611152 PMC2064083

[b38] Pegoraro E. . Congenital muscular dystrophy with primary laminin alpha2 (merosin) deficiency presenting as inflammatory myopathy. Ann. Neurol. 40, 782–791 (1996). 8957020 10.1002/ana.410400515

[b39] Fukada S. . Purification and cell-surface marker characterization of quiescent satellite cells from murine skeletal muscle by a novel monoclonal antibody. Exp. Cell Res. 296, 245–255 (2004). 15149854 10.1016/j.yexcr.2004.02.018

[b40] Urciuolo A. . Collagen VI regulates satellite cell self-renewal and muscle regeneration. Nat. Commun. 4, 1964 (2013). 23743995 10.1038/ncomms2964 PMC3682802

[b41] Uezumi A., Fukada S., Yamamoto N., Takeda S. & Tsuchida K. Mesenchymal progenitors distinct from satellite cells contribute to ectopic fat cell formation in skeletal muscle. Nat. Cell Biol. 12, 143–152 (2010). 20081842 10.1038/ncb2014

[b42] Joe A. W. . Muscle injury activates resident fibro/adipogenic progenitors that facilitate myogenesis. Nat. Cell Biol. 12, 153–163 (2010). 20081841 10.1038/ncb2015 PMC4580288

[b43] Uezumi A. . Identification and characterization of PDGFRalpha+ mesenchymal progenitors in human skeletal muscle. Cell Death Dis. 5, e1186 (2014). 24743741 10.1038/cddis.2014.161 PMC4001314

[b44] Gayraud-Morel B. . A role for the myogenic determination gene Myf5 in adult regenerative myogenesis. Dev. Biol. 312, 13–28 (2007). 17961534 10.1016/j.ydbio.2007.08.059

[b45] Caldwell C. J., Mattey D. L. & Weller R. O. Role of the basement membrane in the regeneration of skeletal muscle. Neuropathol. Appl. Neurobiol. 16, 225–238 (1990). 2402330 10.1111/j.1365-2990.1990.tb01159.x

[b46] Caretti G., Di Padova M., Micales B., Lyons G. E. & Sartorelli V. The Polycomb Ezh2 methyltransferase regulates muscle gene expression and skeletal muscle differentiation. Genes Dev. 18, 2627–2638 (2004). 15520282 10.1101/gad.1241904 PMC525543

[b47] Hughes S. M. . Three slow myosin heavy chains sequentially expressed in developing mammalian skeletal muscle. Dev. Biol. 158, 183–199 (1993). 7687223 10.1006/dbio.1993.1178

[b48] Wigmore P. M. & Stickland N. C. Muscle development in large and small pig fetuses. J. Anat. 137, (Pt 2): 235–245 (1983). 6630038 PMC1171817

[b49] Du M., Yan X., Tong J. F., Zhao J. & Zhu M. J. Maternal obesity, inflammation, and fetal skeletal muscle development. Biol. Reprod. 82, 4–12 (2010). 19516021 10.1095/biolreprod.109.077099 PMC2802110

[b50] Stickland N. C., Widdowson E. M. & Goldspink G. Effects of severe energy and protein deficiencies on the fibres and nuclei in skeletal muscle of pigs. Br. J. Nutr. 34, 421–428 (1975). 1201266 10.1017/s0007114575000487

[b51] Davies B. S. . GPIHBP1 is responsible for the entry of lipoprotein lipase into capillaries. Cell Metab. 12, 42–52 (2010). 20620994 10.1016/j.cmet.2010.04.016 PMC2913606

[b52] Beigneux A. P. . Glycosylphosphatidylinositol-anchored high-density lipoprotein-binding protein 1 plays a critical role in the lipolytic processing of chylomicrons. Cell Metab. 5, 279–291 (2007). 17403372 10.1016/j.cmet.2007.02.002 PMC1913910

[b53] Olafsen T. . Unexpected expression pattern for glycosylphosphatidylinositol-anchored HDL-binding protein 1 (GPIHBP1) in mouse tissues revealed by positron emission tomography scanning. J. Biol. Chem. 285, 39239–39248 (2010). 20889497 10.1074/jbc.M110.171041 PMC2998116

[b54] Basso D. M. . Basso Mouse Scale for locomotion detects differences in recovery after spinal cord injury in five common mouse strains. J. Neurotrauma 23, 635–659 (2006). 16689667 10.1089/neu.2006.23.635

[b55] El-Khodor B. F. . Identification of a battery of tests for drug candidate evaluation in the SMNDelta7 neonate model of spinal muscular atrophy. Exp. Neurol. 212, 29–43 (2008). 18455159 10.1016/j.expneurol.2008.02.025

[b56] Guo L. T. . Laminin alpha2 deficiency and muscular dystrophy; genotype-phenotype correlation in mutant mice. Neuromuscul. Disord. 13, 207–215 (2003). 12609502 10.1016/s0960-8966(02)00266-3

[b57] Menezes M. J. . The extracellular matrix protein laminin alpha2 regulates the maturation and function of the blood-brain barrier. J. Neurosci. 34, 15260–15280 (2014). 25392494 10.1523/JNEUROSCI.3678-13.2014 PMC6608454

[b58] Minasi M. G. . The meso-angioblast: a multipotent, self-renewing cell that originates from the dorsal aorta and differentiates into most mesodermal tissues. Development 129, 2773–2783 (2002). 12015303 10.1242/dev.129.11.2773

