# Supplementary material for: Laminin regulates PDGFRβ+ cell stemness and muscle development
Source: Nat Commun. 2016 May 3;7:11415. doi: 10.1038/ncomms11415 (PMC4857399; doi:10.1038/ncomms11415)
Supplement: Supplementary Figures — 1-12 [file ncomms11415-s1.pdf]

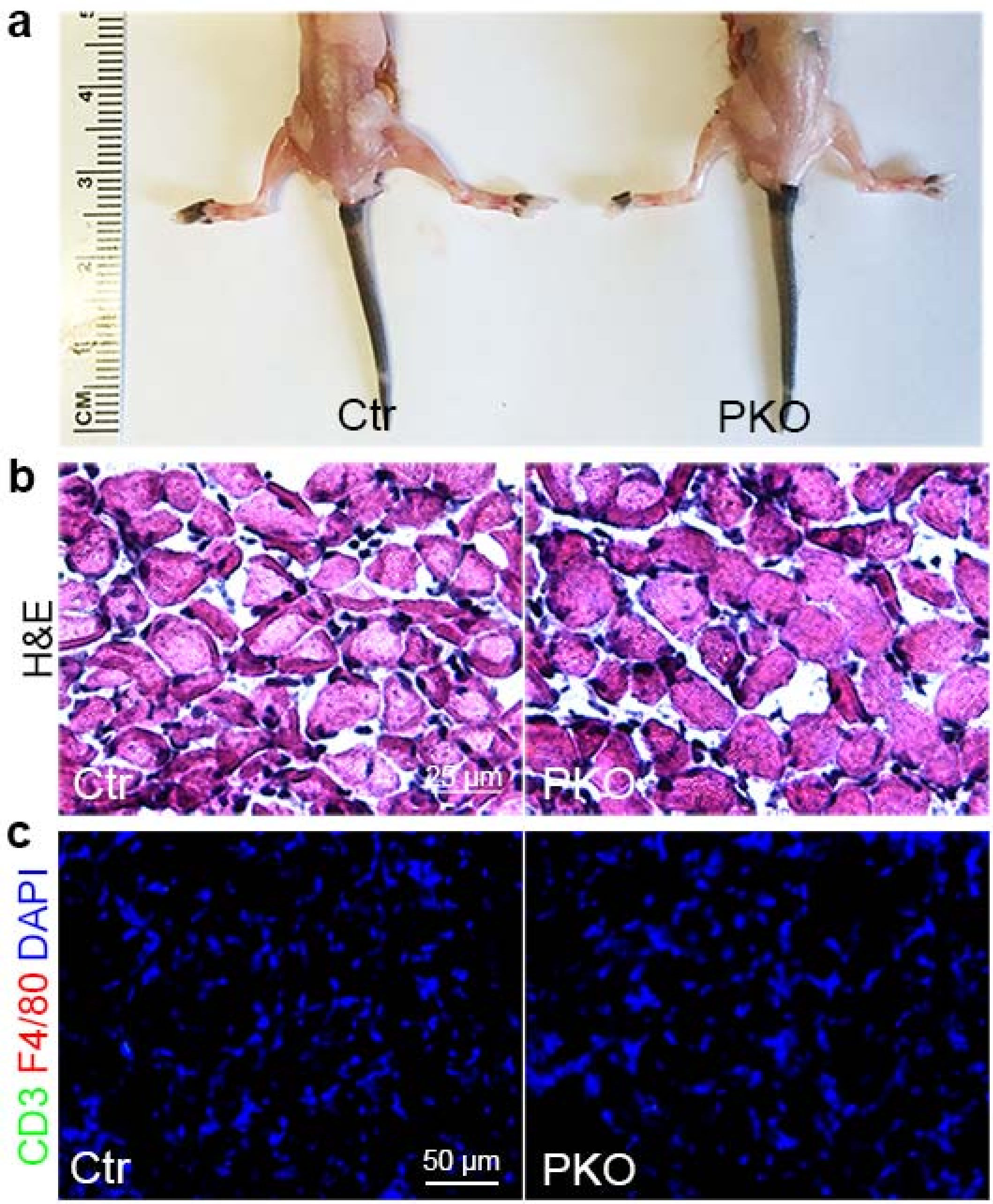

Supplementary Figure 1 Muscle dystrophic phenotype is absent at P6 in PKO mice. (**a**) Size comparison of the P6 control and PKO mice. (**b**) H&E staining of hind-limb muscles from control and PKO mice at P6. (**c**) Immunohistochemical analysis of CD3 (green) and F4/80 (red) in control and PKO muscles at P6. Scale bars represent 25 μm in **b** and 50 μm in **c**.

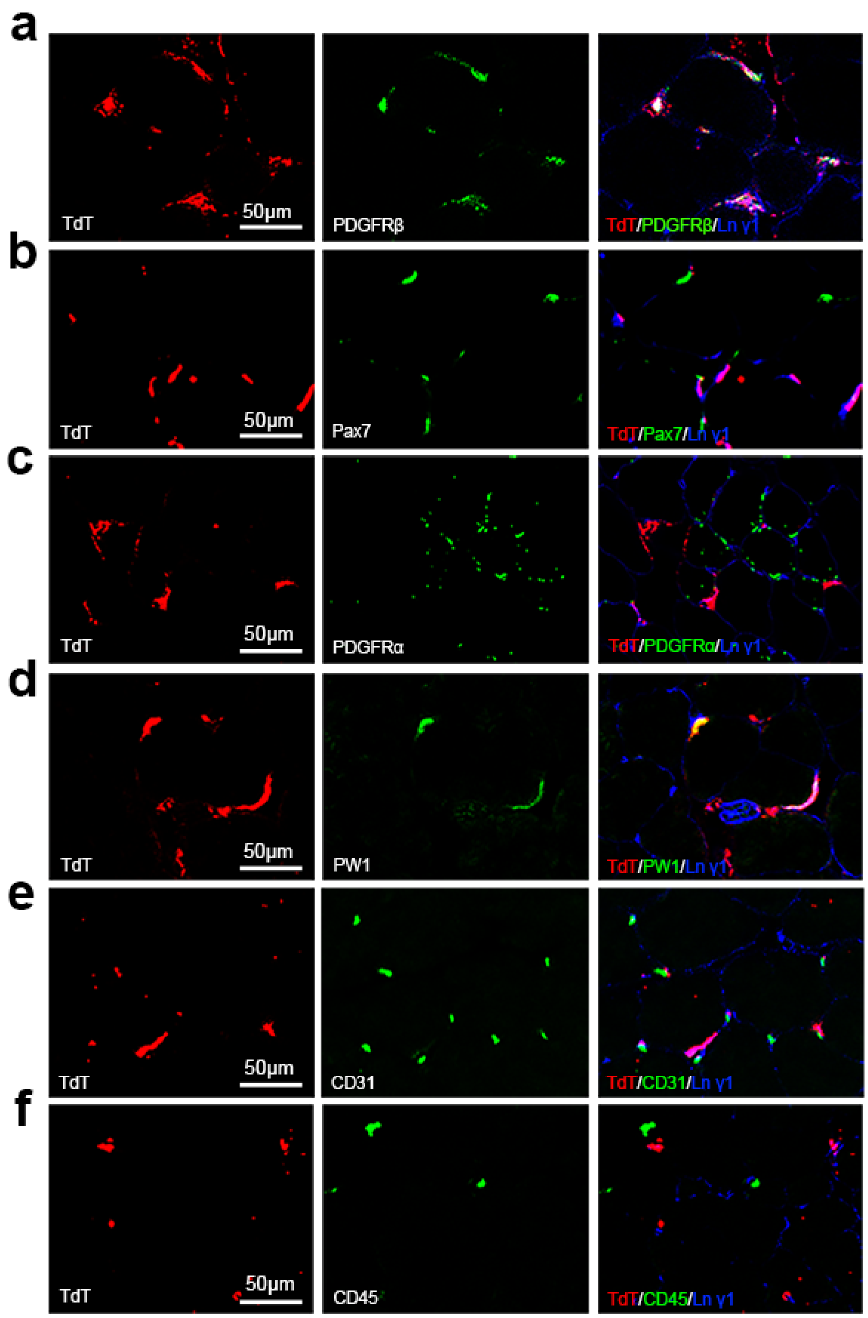

Supplementary Figure 2 Specificity of *Pdgfrβ*-driven *Cre* in injured conditions. Immunohistochemical analyses were performed on muscle sections from laminin-deficient reporter (F/F:Ai14:*Pdgfrβ-Cre*$^{+}$) mice. (**a-f**) TdT (red) expression co-localized with PDGFRβ (**a**, green), PW1 (**d**, green), but not Pax7 (**b**, green), PDGFRα (**c**, green), CD31 (**e**, green) or CD45 (**f**, green) in F/F:Ai14:*Pdgfrβ-Cre*$^{+}$ reporter mice. TdT, tdTomato. Scale bars represent 50 μm.

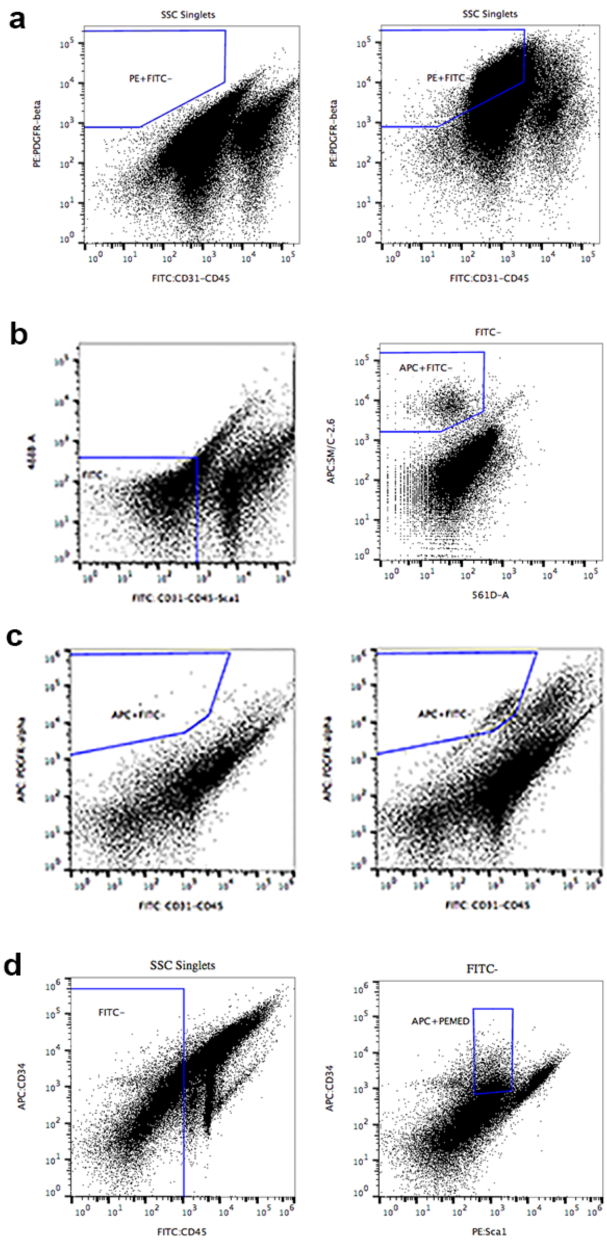

Supplementary Figure 3 Representative FACS plots and gating boundaries. (**a**) Representative FACS plot for PDGFRβ$^+$CD31$^-$CD45$^-$ population. The gating boundary was determined based on PDGFRβ-FMO control. (**b**) Representative FACS plot and gating boundary for SM/C-2.6$^+$CD31$^-$CD45$^-$ Sca1$^-$ cells (satellite cells). (**c**) Representative FACS plot for PDGFRα$^+$CD31$^-$CD45$^-$ population (FAPs). The gating boundary was determined based on PDGFRα-FMO control. (**d**) Representative FACS plot and gating boundary for CD34$^+$Sca1^{Med}CD45$^-$ cells (PICs).

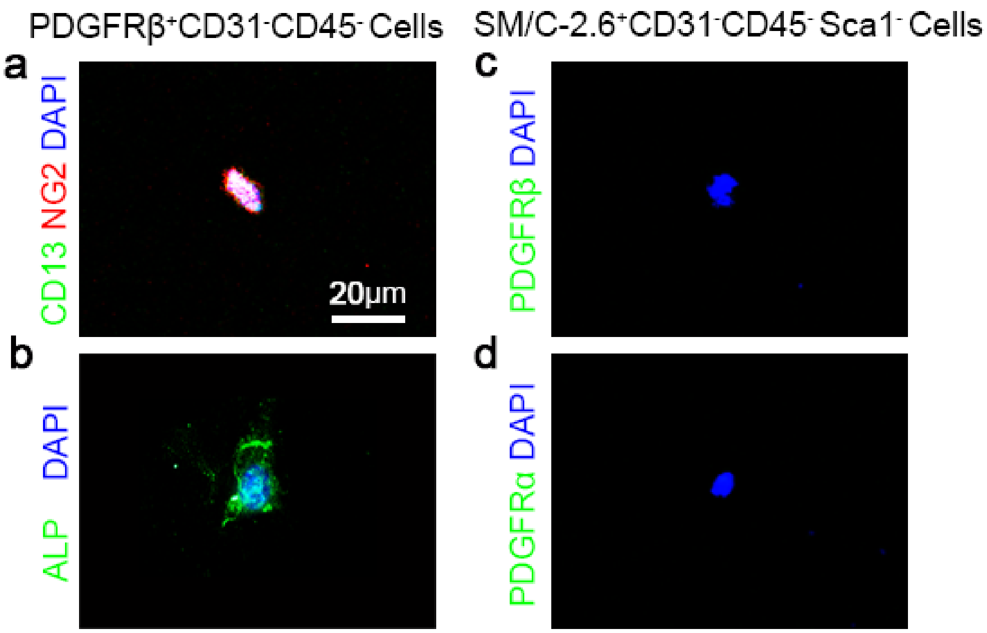

Supplementary Figure 4 Characterization of FACS-isolated PDGFRβ$^+$ cells and satellite cells. (**a**) CD13 (green) and NG2 (red) expression in PDGFRβ$^+$ cells isolated from wild-type mice. (**b**) ALP (green) expression in PDGFRβ$^+$ cells isolated from wild-type mice. (**c** and **d**) PDGFRβ (**c,** green) and PDGFRα (**d,** green) expression in satellite cells isolated from wild-type mice. Scale bar represents 20 μm.

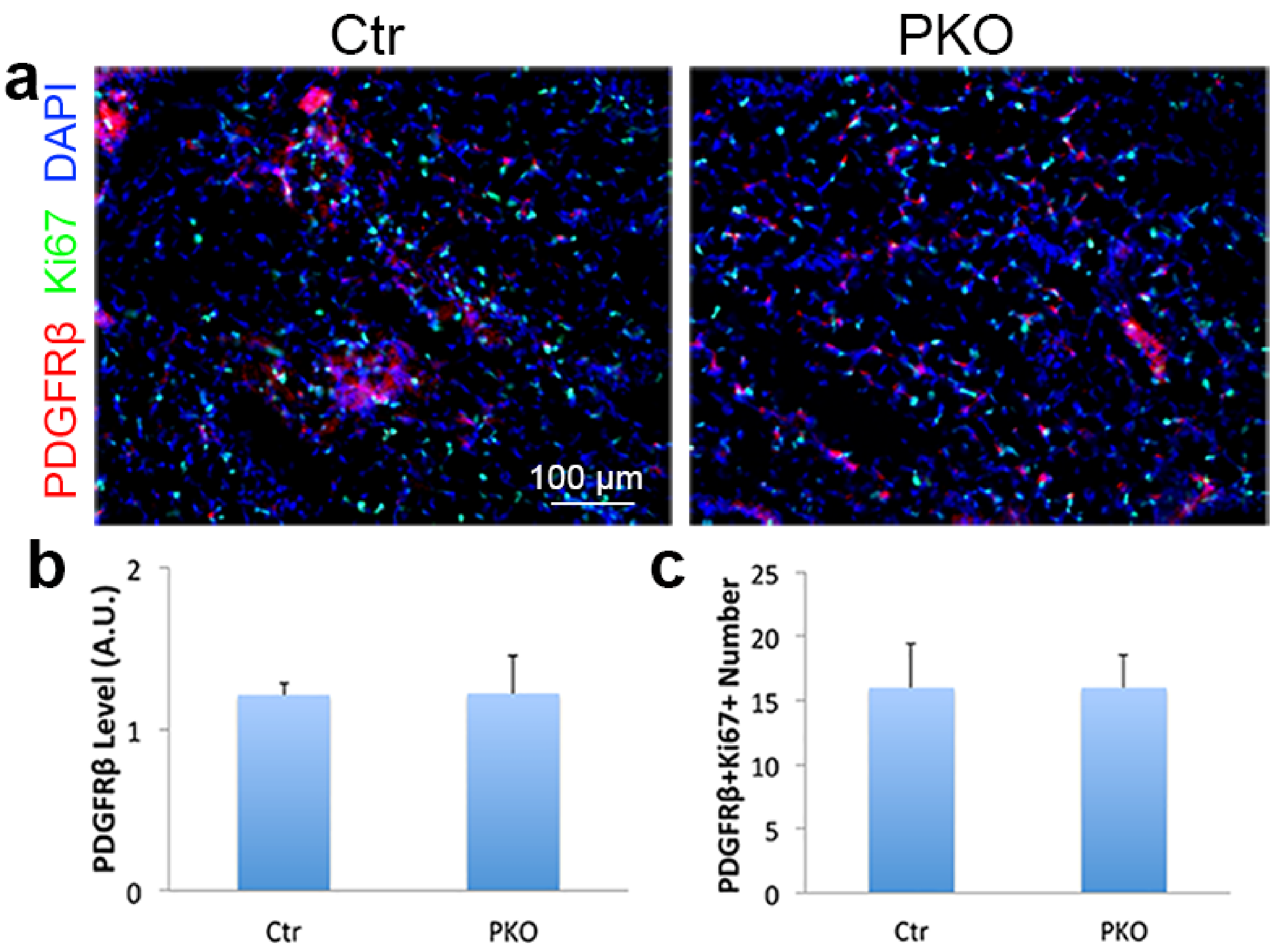

Supplementary Figure 5 The proliferation of PDGFRβ$^+$ cells is unchanged in PKO mice at P6. (**a**) PDGFRβ (red) and Ki67 (green) expression in hind-limb muscles from P6 mice. (**b**, **c**) Quantification of PDGFRβ expression and PDGFRβ$^+$Ki67$^+$ cells in **a**. *n*=3. Scale bar represents 100 μm. Results are shown as mean ± SD.

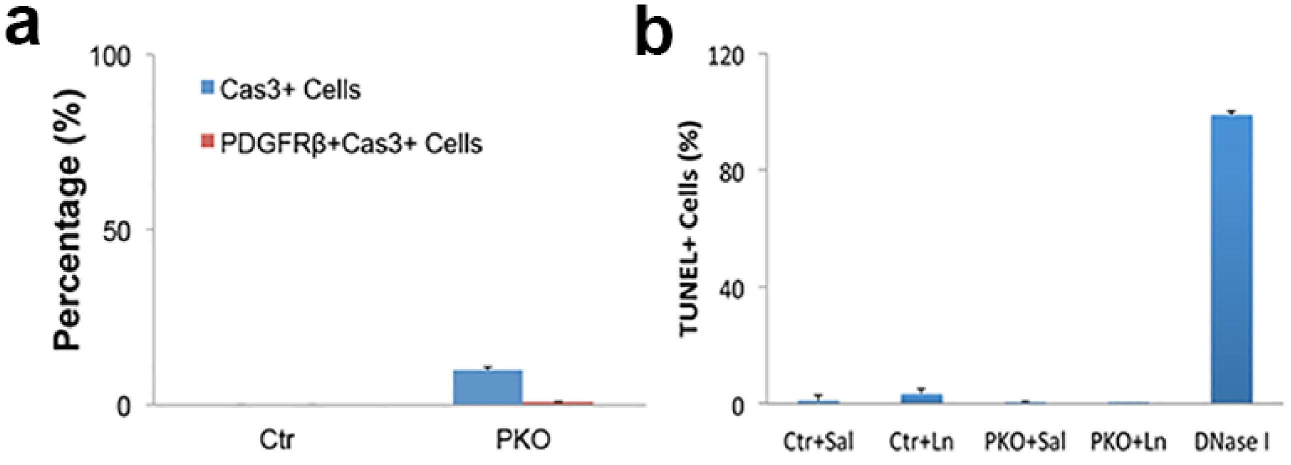

Supplementary Figure 6 Loss of laminin in PDGFRβ$^+$ cells does not induce apoptosis. (**a**, **b**) Quantification of TUNEL$^+$ cells in hind-limb muscles from 2-month-old mice (**a**) and freshly isolated PDGFRβ$^+$ cells (**b**). DNase I-treated PDGFRβ$^+$ cells were used as the positive control. *n*=4. Results are shown as mean ± SD.

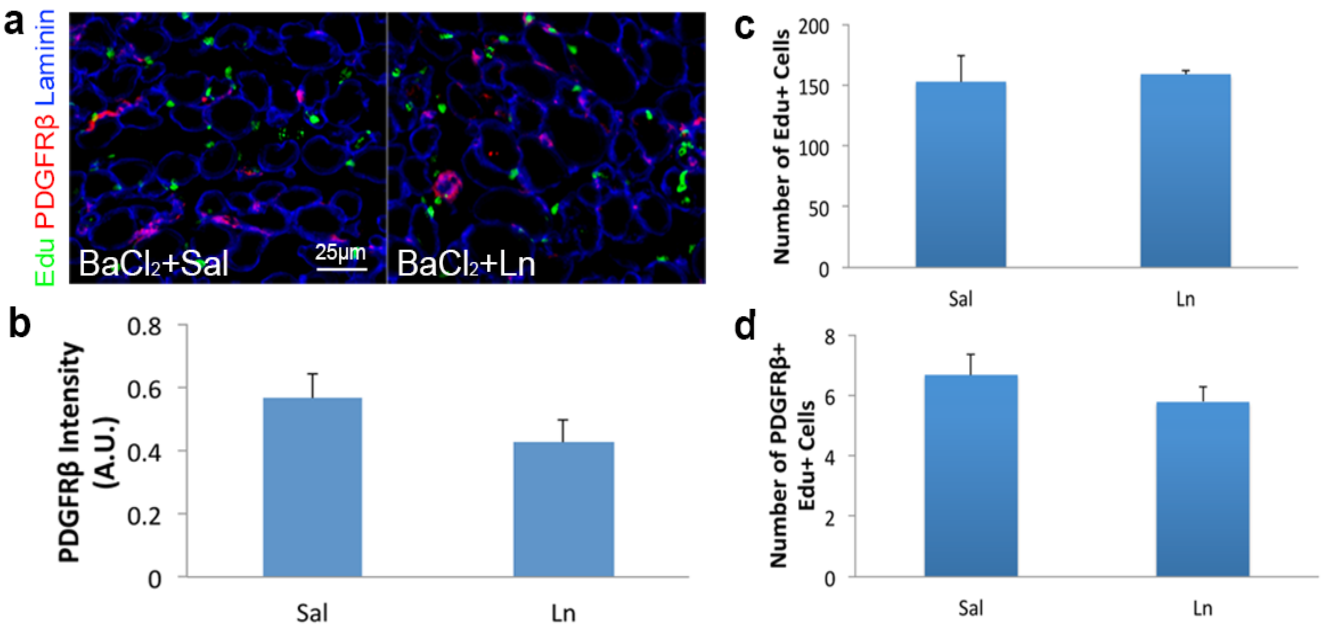

Supplementary Figure 7 The proliferation of PDGFRβ$^+$ cells is unaltered in barium chloride-induced injury. (**a**) Edu (green), PDGFRβ (red), and laminin (blue) expression 5 days after barium chloride injection in the presence of saline or laminin. (**b-d**) Quantification of PDGFRβ expression (**b**), total Edu$^+$ (**c**), and Edu$^+$PDGFRβ$^+$ cells (**d**) in **a**. *n*=3. Scale bar represents 25 μm. Results are shown as mean ± SD.

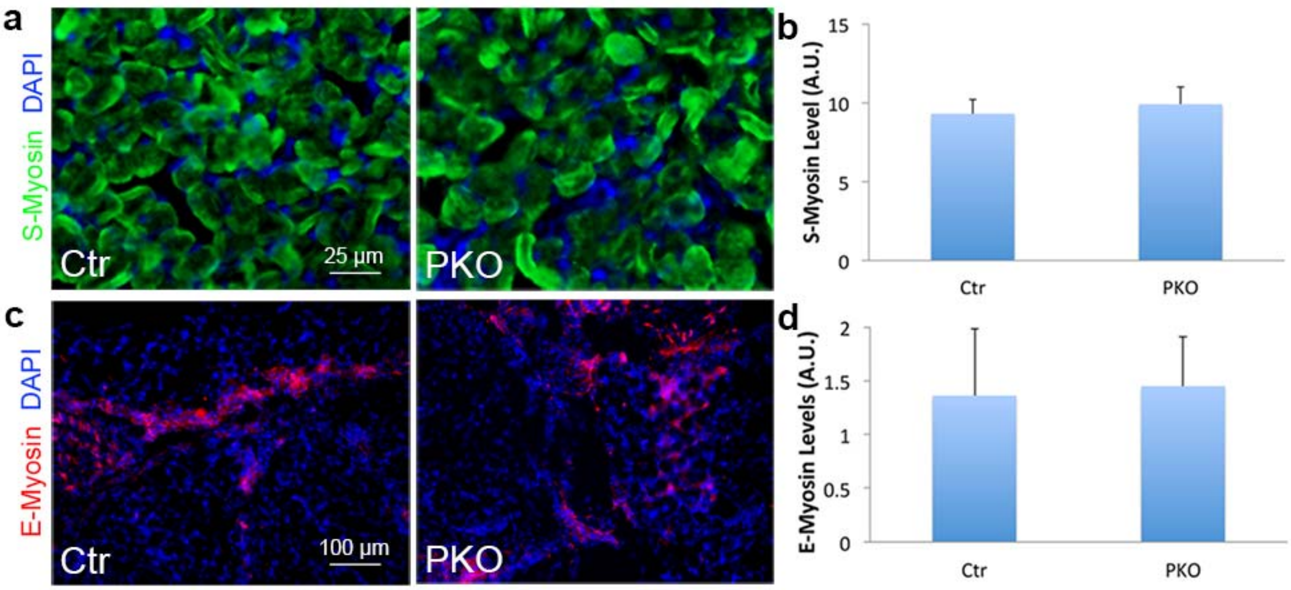

Supplementary Figure 8 The myogenesis of PDGFRβ$^+$ cells is unaffected at P6 in PKO mice. (**a**) S-Myosin (green) expression in hind-limb muscles from P6 control and PKO mice. (**b**) Quantification of S-Myosin expression in **a**. *n*=3. (**c**) E-Myosin (red) expression in hind-limb muscles from P6 control and PKO mice. (**d**) Quantification of E-Myosin expression in **c**. *n*=3. Scale bars represent 100 μm. Results are shown as mean ± SD.

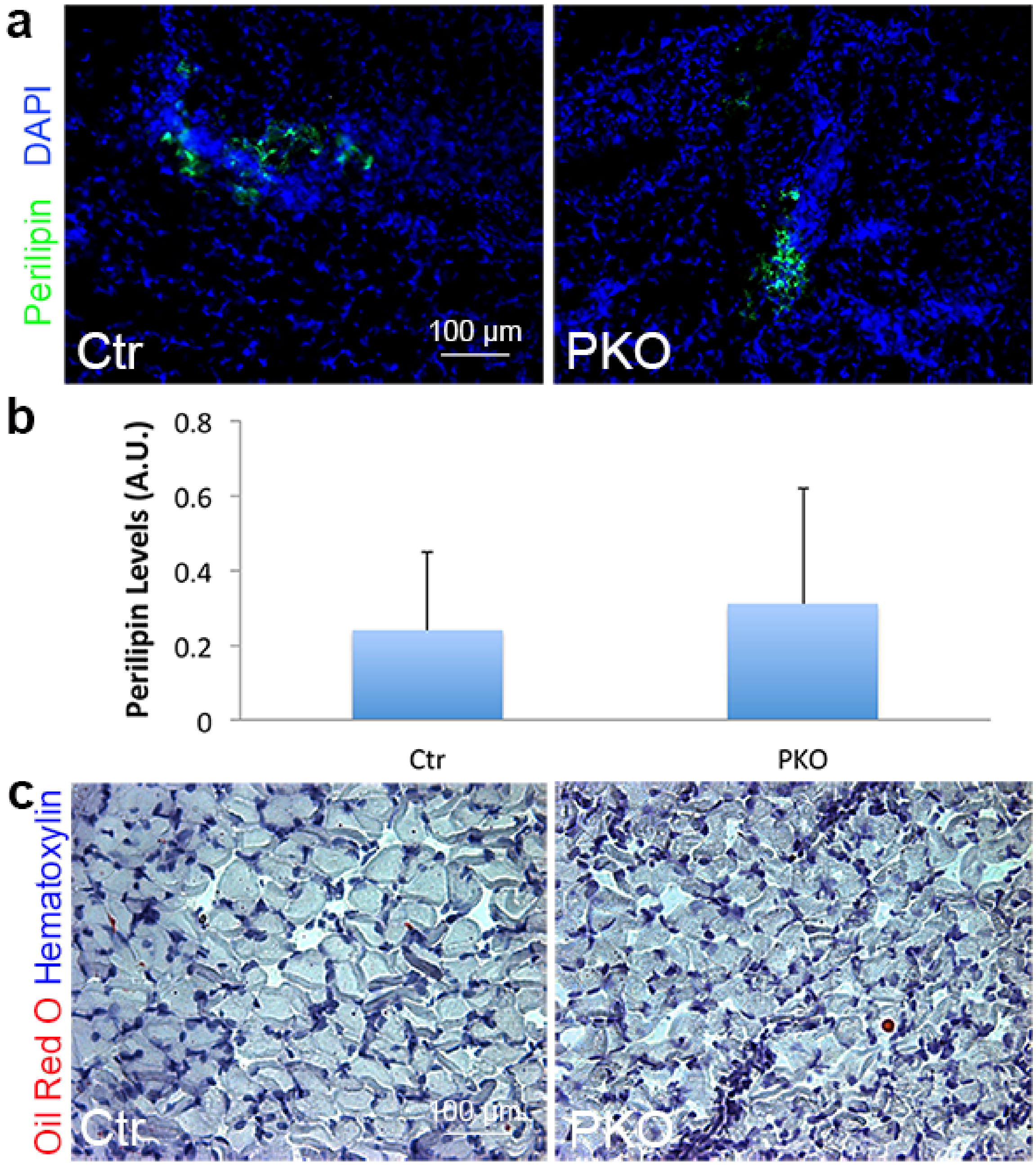

Supplementary Figure 9 The adipogenesis of PDGFRβ^{+} cells is unaffected at P6 in PKO mice. (**a**) Perilipin (green) expression in hind-limb muscles from P6 control and PKO mice. (**b**) Quantification of perilipin expression in **a**. *n*=3. (**c**) Oil Red O staining of hind-limb muscles from P6 control and PKO mice. Scale bars represent 100 µm. Results are shown as mean ± SD.

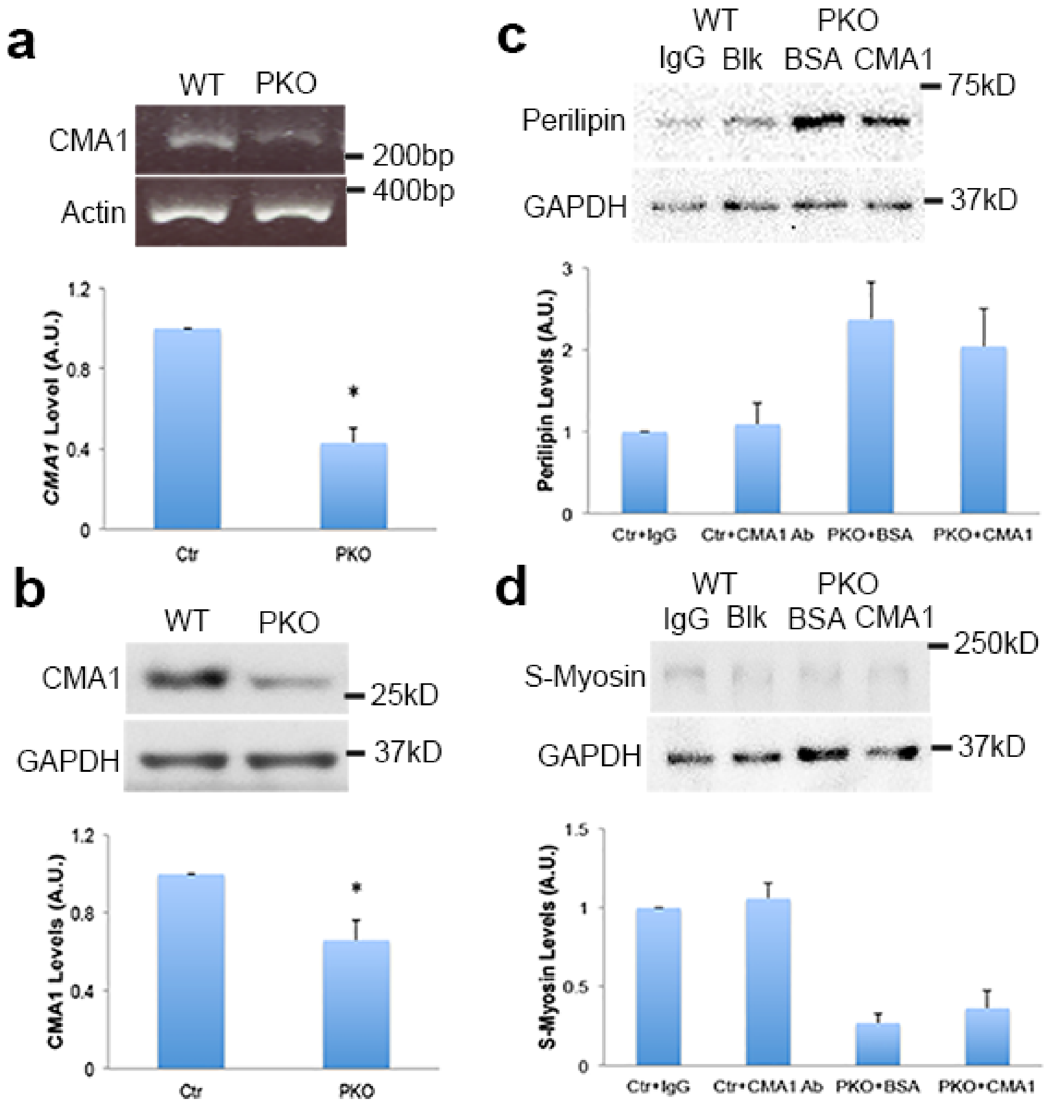

Supplementary Figure 10 CMA1 does not regulate the differentiation of PDGFRβ$^+$ cells. (**a**) RT-PCR analysis of *CMA1* expression in PDGFRβ$^+$ cells. *Actin* was used as a loading control. *n*=3. (**b**) Western blot analysis of CMA1 expression in PDGFRβ$^+$ cells. GAPDH was used as a loading control. *n*=4. (**c**) Western blot analysis of perilipin expression in wild-type PDGFRβ$^+$ cells treated with rabbit IgG or CMA1 antibody and PKO PDGFRβ$^+$ cells treated with BSA or CMA1. GAPDH was used as a loading control. *n*=4. (**d**) Western blot analysis of S-Myosin expression in wild-type PDGFRβ$^+$ cells treated with rabbit IgG or CMA1 antibody and PKO PDGFRβ$^+$ cells treated with BSA or CMA1. GAPDH was used as a loading control. *n*=4. Results are shown as mean ± SD. *p<0.05 (Student's *t*-test).

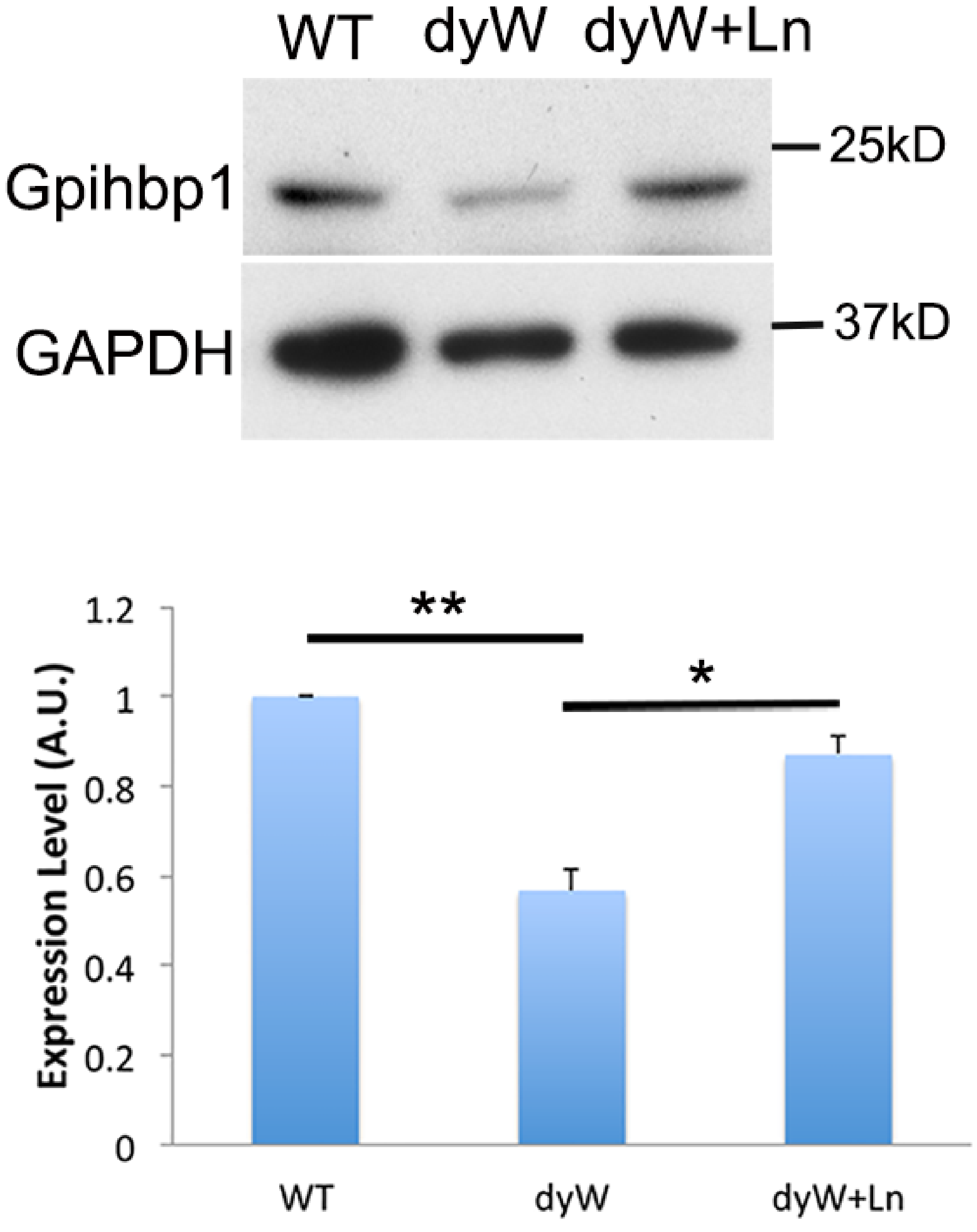

Supplementary Figure 11 Exogenous laminin increases gpihbp1 expression in laminin α2-deficient (dyW) mice. Western blot analysis of gpihbp1 expression in PDGFRβ^{+} cells isolated from wild-type, dyW, or laminin-treated dyW mice. GAPDH was used as a loading control. *n*=3. Results are shown as mean ± SD. **p*<0.05; ***p*<0.01 (Student's *t*-test).

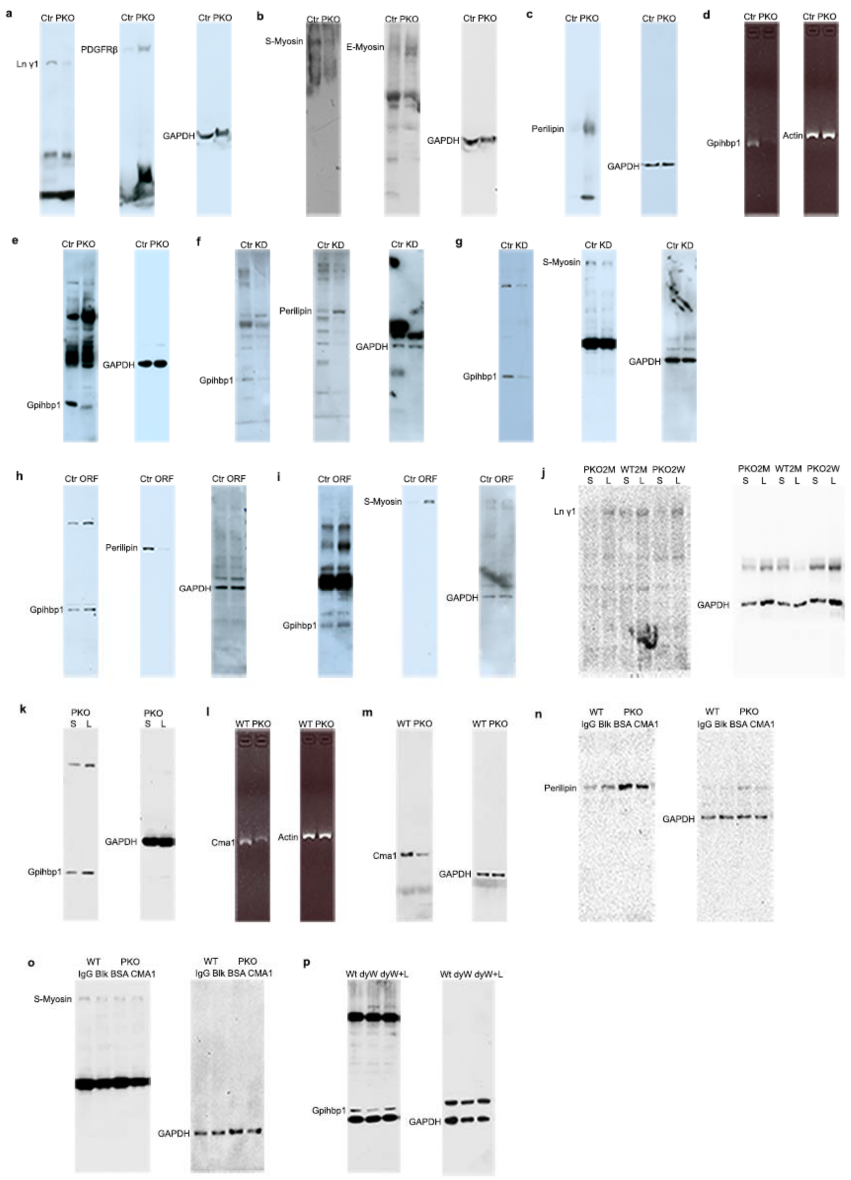

Supplementary Figure 12 Full size images of the blots. (**a**) Full blots for Figure 2g. (**b**) Full blots for Figure 5c. (**c**) Full blots for Figure 6b. (**d**) Full blots for Figure 7d. (**e**) Full blots for Figure 7e. (**f**) Full blots for Figure 7f. (**g**) Full blots for Figure 7g. (**h**) Full blots for Figure 7h. (**i**) Full blots for Figure 7i. (**j**) Full blots for Figure 8f. (**k**) Full blots for Figure 8g. (**l**) Full blots for Supplementary Figure 9a. (**m**) Full blots for Supplementary Figure 10b. (**n**) Full blots for Supplementary Figure 10c. (**o**) Full blots for Supplementary Figure 10d. (**p**) Full blots for Supplementary Figure 11.
